# Unravelling complex interactions during *Toxoplasma*, *Plasmodium*, and *Leishmania* co-infections in French Guiana

**DOI:** 10.1038/s41598-026-40930-8

**Published:** 2026-03-16

**Authors:** Kévin Néron, Constantin Fesel, Magalie Demar, Sylviane Pied

**Affiliations:** 1 Tropical Biome and Immunopathophysiology (TBIP), University of French Guiana, 97300 Cayenne, France; 2 Department of Rheumatology and Clinical Immunology, Medical Center, Faculty of Medicine, University of Freiburg, 79106 Freiburg, Germany; 3 Academic Laboratory of Parasitology and Mycology, Cayenne Hospital, BP 6006, 97306 Cedex Cayenne, French Guiana France; 4 CNRS UMR 9017-INSERM U1019, CIIL-Center for Infection and Immunity of Lille, Institut Pasteur de Lille, University of Lille, 59019 Lille, France

**Keywords:** Protozoan co-infection, Malaria, Cutaneous leishmaniasis, Toxoplasmosis, Cytokine/chemokine response, Integrative analysis, Microbiology, Diseases, Risk factors

## Abstract

*Toxoplasma gondii*, *Plasmodium* spp., and *Leishmania* spp. collectively infect billions and cause millions of deaths worldwide. In French Guiana, the geographical coexistence of malaria, acute toxoplasmosis, and cutaneous leishmaniasis may result in sequential or simultaneous infections. This study assesses the seroprevalence of protozoan multi-infections and their impact on host responses compared to individual infections. We analyzed plasma samples and clinical data from patients with these diseases and healthy controls (*n* = 253) collected at Cayenne Hospital Centre between 2012 and 2022. Seroprevalence for *T. gondii*, *Plasmodium* spp. and *Leishmania* spp. was determined using chemiluminescence and ELISA, and cytokine and chemokine levels were measured via a multiplex assay. Multivariate analysis was utilized to identify distinct biological markers and disease signatures of single and co-infections. About 60% of patients were seropositive for at least two pathogens, with 2.4% having concomitant infections. Significant differences were noted in cytokine profiles, particularly levels of IL-10, IL-17, IL-33, and IFN-α, between single and co-infected patients. These results underline the complex dynamics and high prevalence of co-infections, providing crucial insights for developing effective public health strategies and biomarkers, especially in endemic regions.

**Supplementary Information:**

The online version contains supplementary material available at 10.1038/s41598-026-40930-8.

## Introduction

Vector- and food-borne protozoan infections constitute a significant global health burden and cause extensive morbidity and mortality worldwide. By affecting billions of individuals and being responsible for millions of annual deaths, these infections not only lead to life-threatening conditions but also produce debilitating, long-term effects, such as disfigurement. In endemic regions in particular, individuals frequently encounter a number of protozoan parasites. This exposure may result in a broad spectrum of clinical manifestations, ranging from asymptomatic states to severe, life-threatening diseases^1–6^. Typically, these protozoan infections present as mild or moderately severe illnesses that can trigger inflammatory responses and modulate host immune functions, leading to a state of asymptomatic carriage^7^. The complex interplay between immune responses is critically important for understanding disease dynamics because asymptomatic carriers can either remain resilient (due to effective immune containment, i.e. premunition) or develop a severe disease.

The overall impact of these co-infections on health is usually negative, with exacerbation of the pathogen load and the disease severity. Concomitant or sequential co-infections can lead to intricate immune evasion strategies and altered immune responses to concurrent pathogens; these responses potentially cause severe host damage, including inflammatory conditions, autoimmune disorders, and organ failure^8^. In contrast, previous or concurrent infections might confer heterologous immunity and protect the host against other pathogens. Interindividual variability in responses to these infections can be attributed to genetic, environmental and nutritional factors, the immune history, and the gut microbiota^9^. The intricately intermingled dynamics of host-parasite interactions during co-infections in general (and the impact on parasite fitness and disease outcomes in particular) therefore warrants further investigation.

Malaria (MAL), toxoplasmosis and leishmaniasis are co-endemic in French Guiana^10^. Although these diseases have different vectors, the region’s environmental conditions and lifestyle factors facilitate simultaneous exposure to several parasites, including *Plasmodium* and *Leishmania* species, and *Toxoplasma* strains^11^.

In 2022, MAL accounted for approximately 249 million cases and 608,000 deaths worldwide – mostly in children under the age of five^12^. French Guiana saw a notable decrease in MAL cases between 2005 and 2023, with *P. vivax* being the predominant specie. However, sporadic cases of *P. falciparum* and *P*. *malariae* infections are still observed – particularly in regions where illegal gold mining takes place^13,14^. The patients initially present with fever and anaemia in most cases, with a risk of relapse of *P. vivax* infections due to drug resistance or co-infections^15–19^.

Cutaneous leishmaniasis (CL) is a neglected tropical disease transmitted by the bite of infected female phlebotomine sand flies. It affects 700,000 to 1 million people annually, most of whom live in impoverished regions. The main forms of the disease are characterized by localized or disseminated skin lesions, mucosal lesions or (very rarely) nodular lesions^20^. Over the period 2017–2023, an average of 165 cases per year were detected in French Guiana: these were predominantly caused by *L. guyanensis* (85%), *L. braziliensis* (10%) and, very rarely, *L. amazonensis*, *L. lainsoni* and *L. naiffi*^21^. The prevalence of CL is higher inland and along the borders with Brazil and Suriname^21,22^. In French Guiana, pentamidine is used as the first-line treatment for CL. However, the treatment’s effectiveness is dependent on the *Leishmania* species and the resistance to treatment; for example, resistance to pentamidine isethionate is observed in 5–25% of treated patients and thus remains a significant challenge^23–26^.

Toxoplasmosis caused by *T.* g*ondii* is predominantly a concern for pregnant women and immunocompromised individuals^27^. However, atypical strains in the Amazonian region can cause a severe lung and eye disease (referred to as “Amazonian toxoplasmosis”) in immunocompetent individuals^28–33^. The infection is contracted through consumption of oocyte-contaminated water, undercooked food containing parasitic cysts, blood transfusion, organ transplantation, or vertical transmission. The high seroprevalence of *T. gondii* infection in French Guiana indicates that the co-infection risks might have been underestimated, which might then lead to the emergence of more virulent strains^9,31,34,35^.

**Fig. 1:**
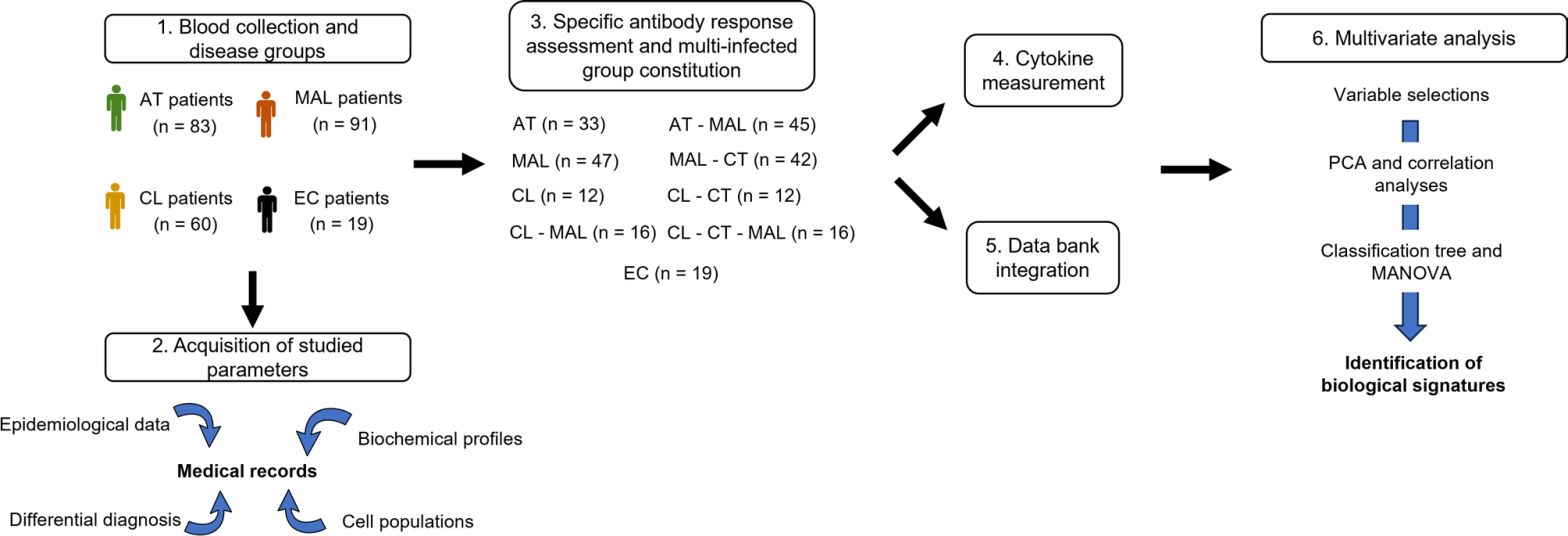
The study design and the study cohort. (1) Patients were included based on their infection with toxoplasmosis, malaria or leishmaniasis or their inclusion in the EC group. (2) Parasitic factors, positivity to other pathogens and all studied parameters were acquired from medical records. All patients with concomitant infection with *T. gondii*,* Plasmodium* spp. or *Leishmania* spp. (6) or outlier data (5) were excluded from the multivariate analyses. (3) Specific antibody responses to *T. gondii*, *Plasmodium* spp. or *Leishmania* spp. were assessed by CMIA or ELISA and the 4 disease groups were then divided into 9 subgroups according to their seropositivity. (4) Then inflammatory responses were assessed by flow cytometry in a multiplexed assay and (5) data were integrated for multivariate analysis. (6) Parameters discriminating between the 3 diseases were selected by bivariate analysis, PCA and correlation analysis. Parameters discriminating the infection subgroups were further determined using classification trees, and the significance of these parameters was assessed using MANOVA. Finally, biological signatures discriminating infection groups were proposed in a model. AT, acute toxoplasmosis; MAL, malaria; CL, cutaneous leishmaniasis; EC; endemic control.

The present study was designed to assess the prevalence and impact of co-infections among *Plasmodium*, *Toxoplasma*, and *Leishmania* in French Guiana. The objectives were to identify distinct biochemical and cytokine/chemokine profiles in individuals with single infections and co-infections and, within the context of translational medicine, to contribute to a deeper understanding of the dynamics of co-infection, disease progression, and host-parasite interactions (Fig. 1).

## Results

### Characteristics of the study population

**Fig. 2:**
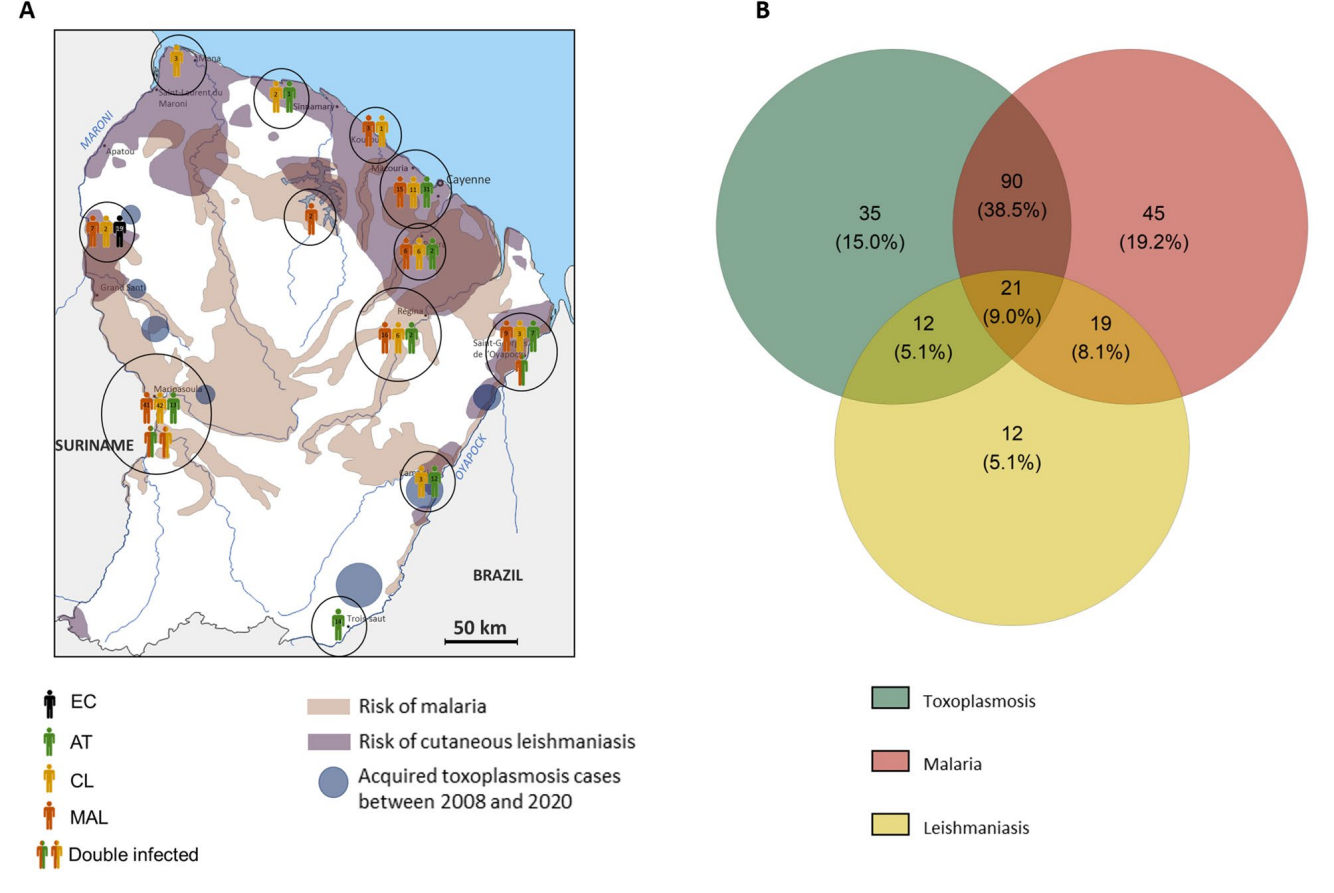
The geographical distribution and risk of protozoan multi-infections in French Guiana. **A** Geographical distribution of patients infected with *P. vivax*, *P. falciparum*, *L. guyanensis*, *L. braziliensis* and/or *T. gondii*. The black circles are not proportional to the size of the population included in these areas. The map was created based on the malaria risk map for French Guiana (2018) from the Agence Régionale de Santé de la Guyane, and adapted from the maps by Jagadesh et al. (2021) and Labaudinière et al. (2017). **B** A Venn diagram showing the seroprevalences of *T. gondii*, *Plasmodium* spp. and *Leishmania* spp. in the study cohort, including both acute and chronic infections.

We included 253 patients (157 males and 86 females; male-to-female ratio: 1.72) living in the Maripasoula and Saint-Georges de l’Oyapock areas of French Guiana or in the coastal regions bordering Suriname and Brazil (Fig. 2A; Table 1). In the Maripasoula region, Brazilians account for a third of the population. The median (range) age of the participants was 34 years (18–82). There were 83 patients in the AT group (50 with MT and 33 with ST), 91 in the MAL group, and 60 in the CL group. Notably, six of the patients presented with concurrent infections: three with AT + MAL, and three with CL + MAL. Serological testing revealed that approximately 60% of the patients were positive for at least two of the three parasites studied (Fig. 2B).

**Table 1:** Characteristics of the study population

Disease	Acute acquired toxoplasmosis		Malaria				Leishmaniasis					Endemic control
Species/strain	South America *T. gondii* strains		*P. vivax*		*P. falciparum*		*Leishmania sp.*	*L. guyanensis*			*L; braziliensis*	None
Clinical outcome	MT	ST	MM	SM	MM	SM	LCL	LCL	DL	MCL	LCL	None
Number of patients (%)	50 (60.30)	33 (39.70)	78 (85.71)	5 (5.50)	6 (6.59)	2 (2.20)	4 (6.67)	44 (73.33)	3 (5.00)	1 (1.67)	8 (13.33)	19 (100)
Median age (range)	35 (20–71)	35 (18–64)	33 (18–66)	29 (22–82)	37 (23–58)	36 (22–49)	47 (31–50)	30 (18–56)	47 (31–62)	33	36 (18–42)	33 (23–60)
Sex (M/F)	29/21	20/13	48/30	3/2	4/2	2/0	1/3	32/12	3/0	0/1	5/3	17/2
Blood collection season (No.)	Long rainy (37)	Long rainy (17)	Dry (47)	Dry (2)	Dry (4)	–	Short summer of March (2)	Short rainy (21)	Short rainy (2)	Long rainy (3)	Short rainy (3)	Dry (19)
Main ethnic group (No.)	Amerindian (13)	Amerindian (8)	Brazilian (32)	Caucasian (2)	African (3)	–	Brazilian (2)	Brazilian (27)	Brazilian (32)	Brazilian (2)	Brazilian (3)	–
PCR (positive/total)	5/10	11/15	19/20	–	2/2	–	0/3	3/4	2/2	1/1	3/3	None
Malaria parasitaemia, median (range)	–	0.01	0.08 (0.01–1)	0.17 (O.01–0.64)	1.7 (0.05–1.7)	1.51 (1.4–1.62)	–	0.34 (0.28–0.4)	–	–	–	–
Direct microscopy examination (positive/total)	–	–	77/85	5/5	5/6	2/2	0/2	36/43	1/2	1/1	4/7	–
Cutaneous lesions, median (range)	–	–	–	–	–	–	1.5 (1–3)	1 (1–14)	12.5 (10–15)	5	1 (1–4)	–
Type of lesions (ulcer/nodule/both)	–	–	–	–	–	–	2/0/2	26/0/6	1/0/1	1/0/0	5/0/1	–
IgM anti–7 g level, median (range)	12.6 (2.5–36.1)	19.6 (0.7–37)	0.18 (0.05–8.81)	0.17 (0.6–1.22)	0.23 (0.13–0.41)	0.24 (0.22–0.25)	0.12 (0.1–0.15)	0.15 (0.06–0.45)	0.26	0.19	0.14 (0.07–0.54)	–
IgG anti–7 g level, median (range)	70.4 (0.1– > 2000)	36.3 (0–1416.2)	6.3 (0–962.5)	5.3 (0.1^14.7)	0.95 (0.03–50.2)	2.65 (0.1–5.2)	1.5 (0.1–5.8)	14.2 (0.31–198.9)	162.8	8.1	0.55 (0.1–66)	–
Total Ig anti–P. *sp.* or malaria history declared (positive/total)	22/48	22/33	29/39	1/1	4/4	1/1	3/4	27/42	1/3	1/1	1/4	12/19
Total number of patient	83		91				60					19

Specifically, 50.5% (*n* = 46) of the patients with MAL and 51.7% (*n* = 31) of patients with CL had prior infections with *T. gondii*, suggesting the presence of chronic toxoplasmosis (CT); these patients respectively constituted the sequential co-infection groups MAL - CT and CL - CT. Furthermore, 57.8% (*n* = 48) of the patients in the AT group and 60.0% (*n* = 36) of the patients in the CL group were positive for antibodies to either *P. falciparum* or *P. vivax* antigens, indicating sequential co-infections with MAL. These individuals were categorized respectively into AT - MAL and CL - MAL groups. Using a multinomial logistic regression test, the likelihood of being classified in the AT - MAL group was significantly higher during the dry season and the short rainy season, with odds ratios [95%CI] of 0.14 [0.02; 0.93] (*p* = 0.041) and 0.06 [0.08; 0.57] (*p* = 0.014), respectively. Furthermore, our data show that 9.0% of the patients had been in contact with all three parasites and so were included in the CL - CT - MAL group. Patients exposed solely to AT, MAL or CL constituted the AT, MAL and CL groups, respectively.

### Liver dysfunction markers as discriminants of the type of protozoan disease

**Fig. 3:**
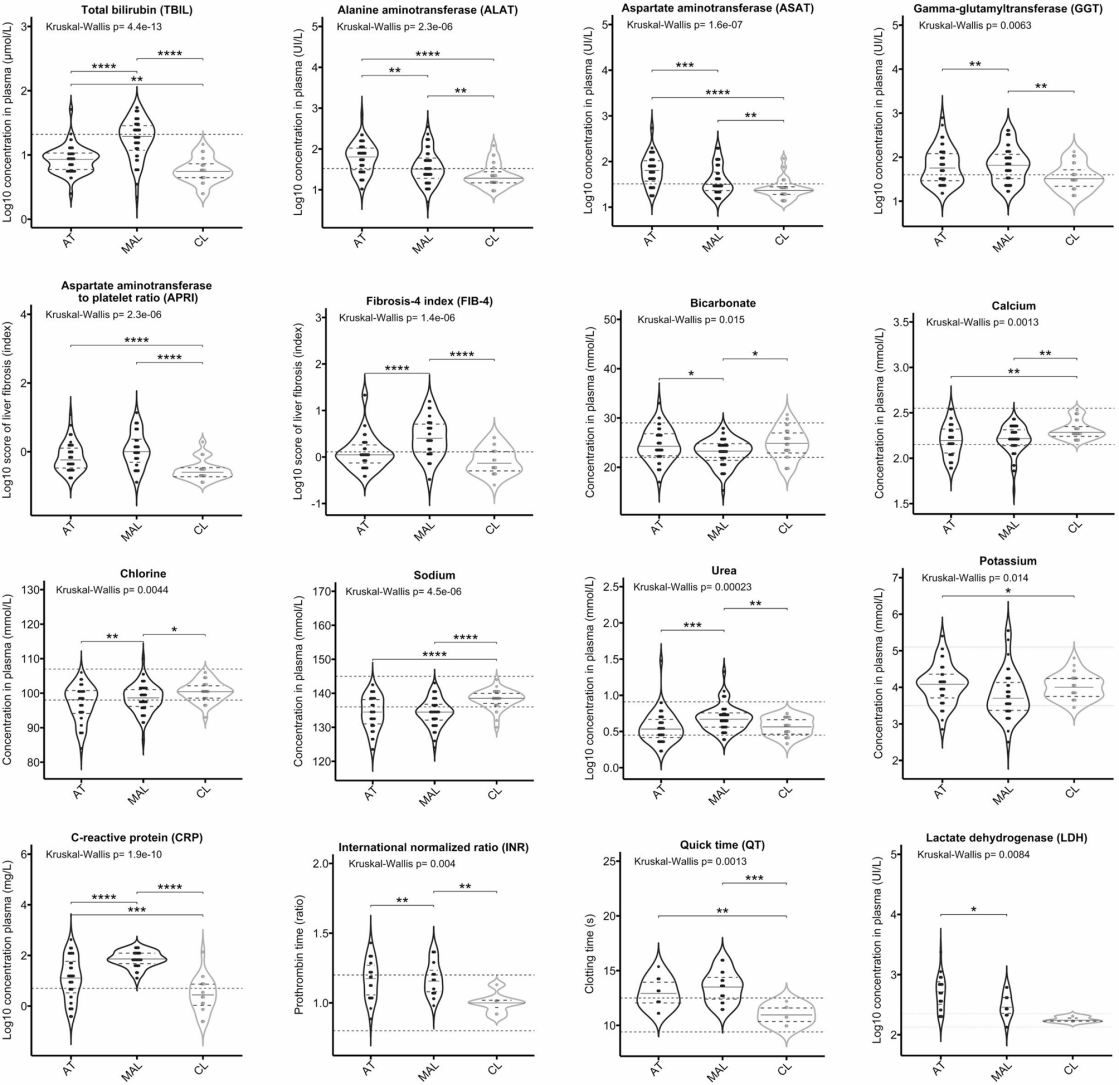
Biochemical profile. Violin plots show the median and quartiles. The dashed lines indicate the standard clinical reference values for each variable. The significance level in a Conover post-hoc test is denoted as ****: *p* < 0.0001; ***: *p* < 0.001; **: *p* < 0.01; and *: *p* < 0.05.

We next looked at whether the plasma levels of 30 biochemical analytes enabled us to distinguish between patients with each of the three protozoan diseases (Fig. 3). Sixteen of these variables differentiated significantly groups. Notably, the fibrosis-4 index (FIB-4) and levels of total bilirubin (TBIL), urea, and C-reactive protein (CRP) were higher in the MAL group than in the AT group. In contrast, levels of ASAT and ALAT were markedly higher in the AT group than in the MAL group. The values in the CL group were close to control values.

**Fig. 4:**
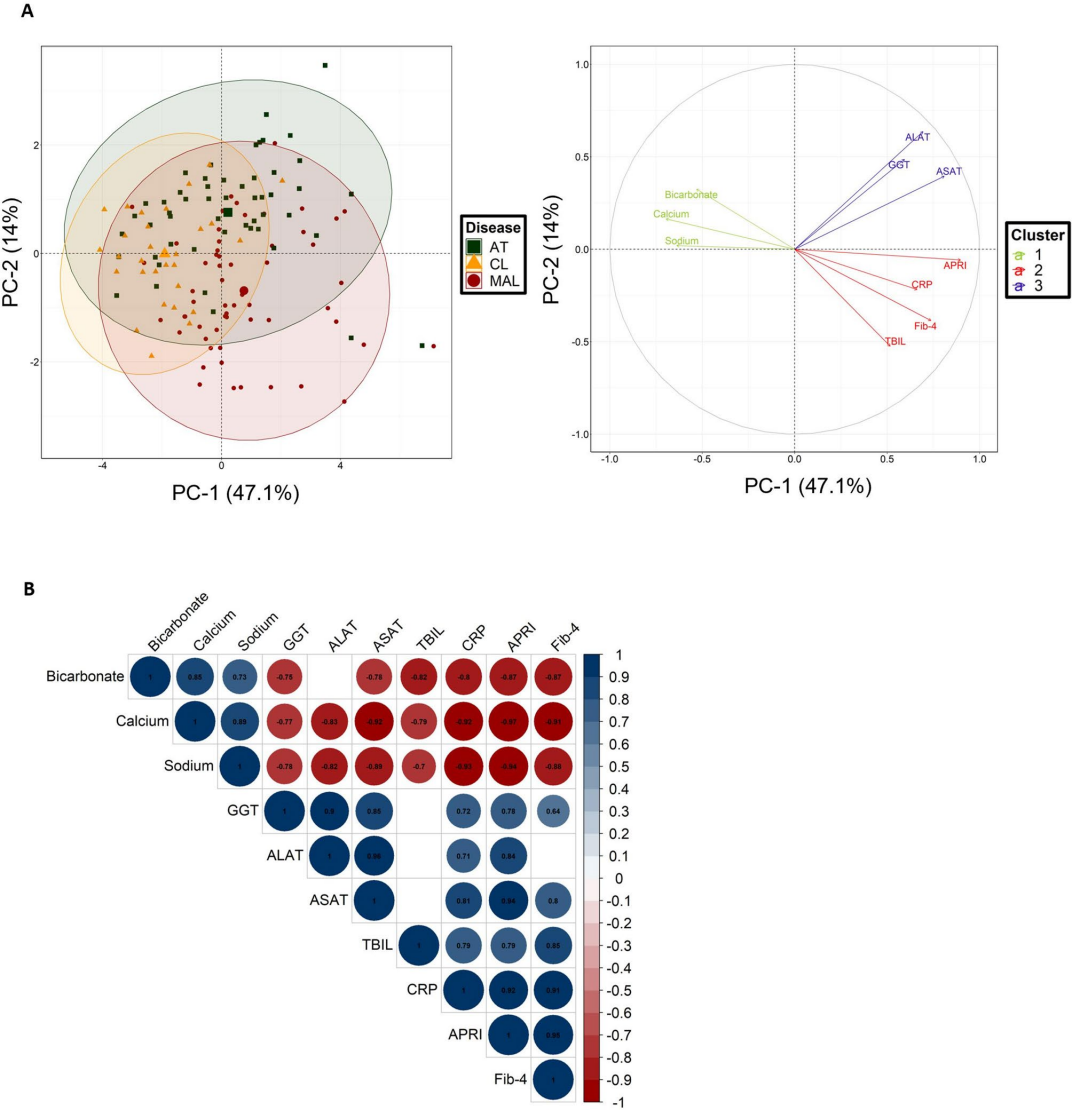
Discriminant biochemical variables. **A** A principal component analysis (PCA) showing individuals (left graph) and the variable factor loads (right graph) for the first two principal components, which with explained 61.1% total variance. Variables with small contributions to the principal component and patients with more than five missing values were excluded from the analysis. The remaining data were imputed by the mean of the variable. To distinguish between the disease groups, three clusters were determined using a *k* means algorithm. **B**. A Spearman correlogram of the biochemical variables that contributed the most to the PCA. Only correlations with *p* < 0.01 are shown. The size of the spot is proportional to the strength of the correlation, and the colour of the spot corresponds to the direction (blue for a positive correlation and red for a negative correlation). The value of the correlation coefficient is noted in the spot, and the scale is given on the right.

A PCA provided an overview of the distribution of patients with regard to the biochemical markers. After filtering data based on missing values and selecting variables for their contribution to the principal components, we observed that the 10 analytes grouped the patients (*n* = 140) into three moderately separate disease clusters (Fig. 4A). However, none of the variables clearly differentiated between the CL group and the other groups. Cluster 2 (including the aspartate aminotransferase-to-platelet ratio index (APRI), FIB-4, TBIL, and CRP) partially distinguished between the MAL group and the AT group. Cluster 1 (comprising the electrolytes bicarbonate, calcium and sodium) and cluster 3 (comprising the hepatic variables ALAT, gamma-glutamyl-transferase (GGT), and ASAT) did not discriminate significantly between the patient groups.

The MAL group was characterized by elevated levels of hepatic cytolysis markers, whereas the AT group presented elevated plasma transaminase levels. A Spearman correlogram covering all patients (Fig. 4B) revealed two distinct correlation blocks: one indicated positive correlations between liver dysfunction markers, and the other showed negative correlations between liver dysfunction markers and the plasma electrolytes (bicarbonate, calcium, and sodium).

We further examined the impact of these factors in the various clinical groups (Supplementary Fig. S1). Significant differences between the MT and ST groups were observed, with higher levels of liver dysfunction markers and lower levels of electrolytes in the ST group (Supplementary Fig. S1A). The titre of IgM anti-*Tg*SAG1 was negatively correlated with plasma sodium, bicarbonate and calcium levels and positively correlated with ALAT and ASAT levels. The IgM anti-*Tg*SAG1 titre was higher in the ST group than in the MT group (Supplementary Fig. S1B). The levels of direct bilirubin and CRP were higher in the SM group than in the MM group, while the level of calcium was lower (Supplementary Fig. S2A). Parasitaemia was positively correlated with TBIL but not correlated with disease severity (Supplementary Fig. S2B). In the CL group, we did not observe any significant correlations between the number of skin lesions and biochemical factors. However, multiple linear regression analysis showed an influence of sex on electrolytes (calcium, bicarbonate, sodium) and CRP (lower in females). FIB-4 increased with age (Supplementary Table 2). Taken as a whole, these findings suggest that liver dysfunction markers reflect complications arising during AT or MAL infections and during the chronic phase of CL.

### Lymphocyte and innate immune cell profiles, as a function of the type of parasite infection

**Fig. 5:**
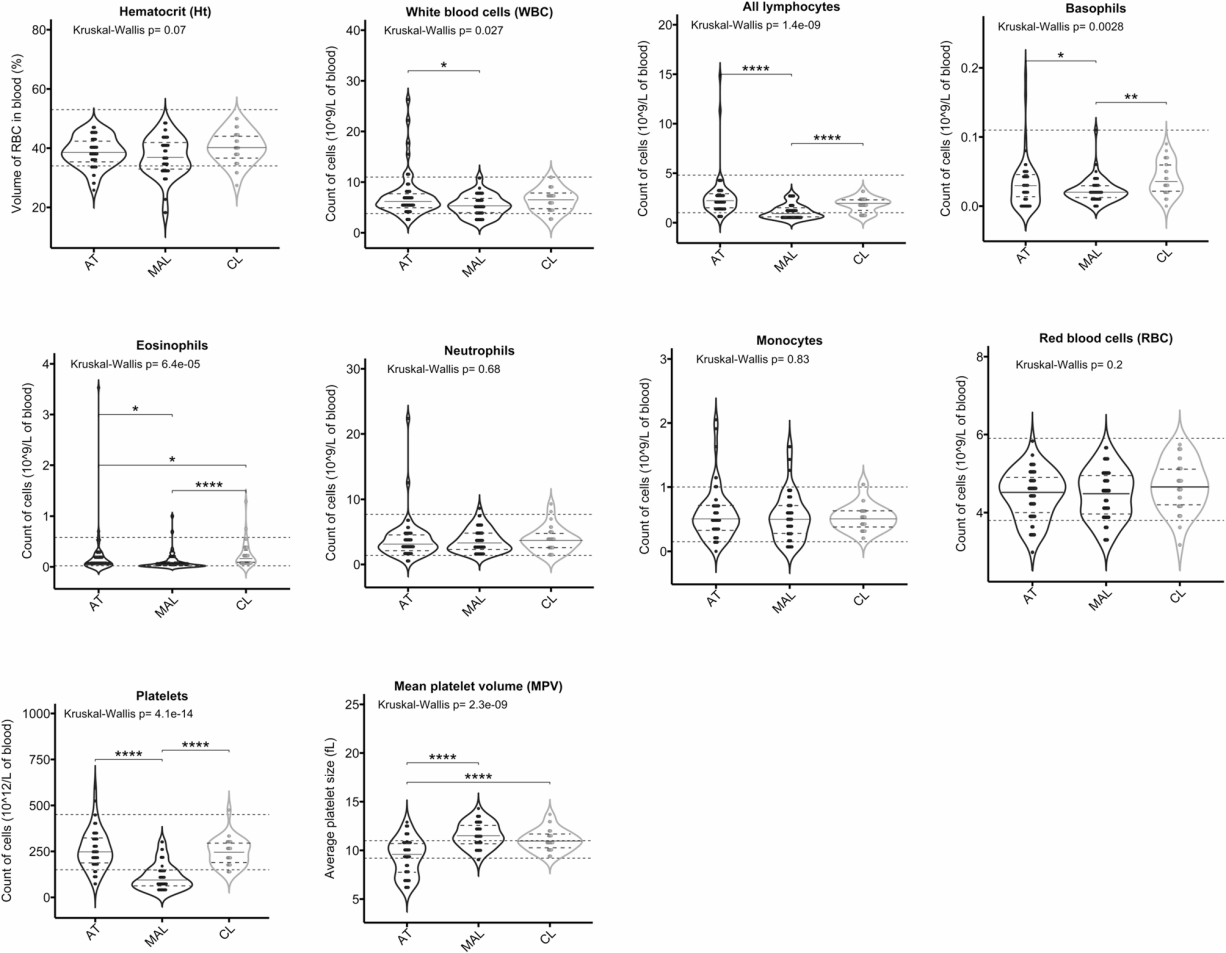
Discriminant cell populations. Violin plots show the median and quartiles. The dashed lines indicate the standard clinical reference values for each variable. Each point corresponds to an individual. The significance level in a Conover post-hoc test is denoted as ****: *p* < 0.0001; **: *p* < 0.01; and *: *p* < 0.05.

We next evaluated the adaptive and innate immune cell responses in the different infection groups, with a focus on granulocyte (basophil/eosinophil/neutrophil), monocyte and total lymphocyte counts. According to violin plots (Fig. 5), the lymphocyte, basophil, eosinophil, and platelet counts were higher in the AT and CL groups than in the MAL group. The mean platelet volume (MPV) was significantly lower in the AT group than in the two other groups. The MPV was similar in the MAL and CL groups. In the latter, linear regression showed that MPV tended to be influenced by the number of skin lesions (Supplementary Table 2). The three groups did not differ significantly with regard to the red blood cell count or the haemoglobin level. These subpopulation distributions suggest that the antiparasite innate response is stronger in AT and CL than in MAL, with less pronounced thrombocytopenia.

### Cytokine clusters differentiate between disease groups

**Fig. 6:**
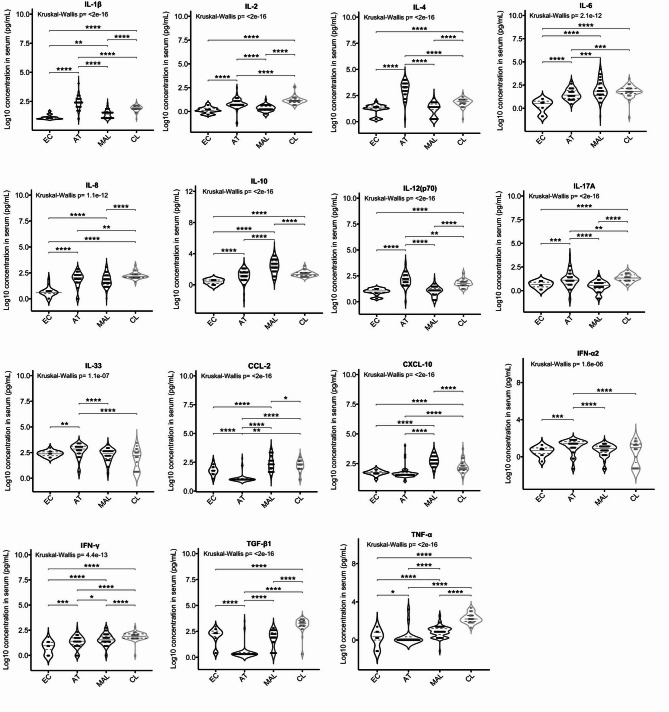
Cytokine spectra. Violin plots show the median and the quartiles. The dashed lines indicate the standard clinical reference values for each variable. Each point corresponds to an individual. The significance level in a Conover post-hoc test is denoted as ****: *p* < 0.0001; ***: *p* < 0.001; **: *p* < 0.01; and *: *p* < 0.05.

**Fig. 7:**
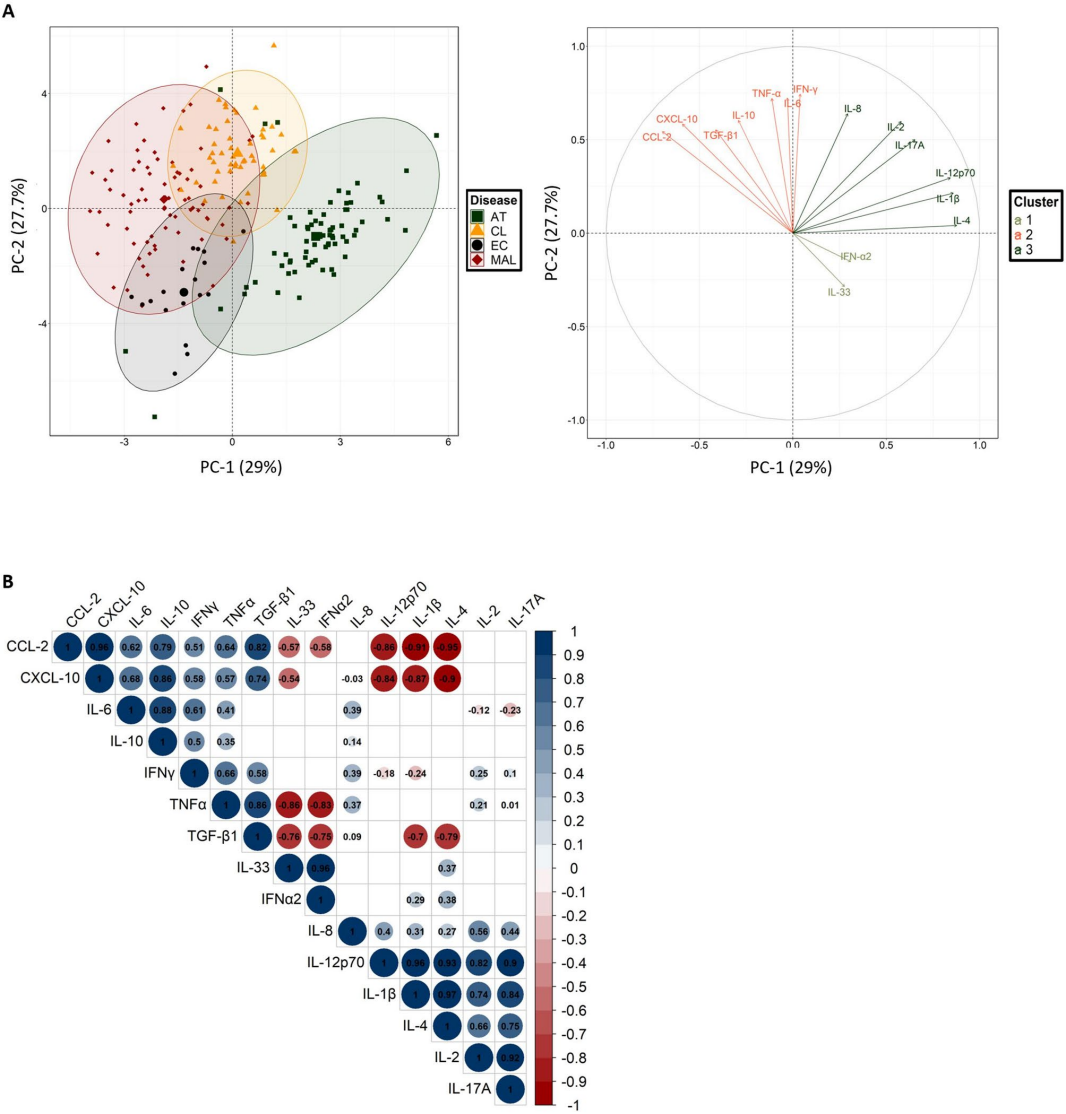
Discriminants for the inflammatory response in each disease group. **A** Principal component analysis of individuals (left graph) and the variable correlations (right graph) for the first two principal components (accounting for 56.7% of the total variance). Patients with more than 6 missing values were excluded from the analysis. Missing data were imputed with the mean. To distinguish between the disease groups, four clusters were determined using a *k* means algorithm. **B** A Spearman correlogram. Only correlations with *p* < 0.01 are shown. The size of the spot is proportional to the strength of the correlation, and the colour of the spot corresponds to the direction (blue for a positive correlation and red for a negative correlation). The value of the correlation coefficient is noted in the spot, and the scale is given on the right.

We next assayed levels of 15 cytokines and chemokines in plasma samples from 200 infected patients and 19 ECs. Significant differences between disease groups and ECs were observed (Fig. 6). A PCA of cytokine/chemokine levels revealed four clusters that distinguished between MAL and AT patients but did not distinguish these groups from the CL group. Notably, the AT group predominantly exhibited elevated levels of IL-33 and IFN-α2 (cluster 1) and IL-1β, IL-2, IL-4, IL-8, IL-12(p70), and IL-17 A (cluster 3) whereas the MAL group was characterized by elevated levels of CXCL-10, CCL-2, IL-10 and TGF-β1. The CL group showed a mixed profile, with elevated levels of TGF-β1, TNF-α, IL-6, IL-10, CCL-2, CXCL-10, and IFN-γ (cluster 2) and IL-8 (Fig. 7A). The EC group showed relatively low levels of all cytokines except for IL-33, CXCL-10, CCL-2, and TGF-β1 (Figs. 6 and 7A).

A Spearman’s correlation analysis (Fig. 7B) revealed strong associations within two blocks of positively correlated cytokines/chemokines that specifically differentiated between the MAL and AT groups. IL-10 did not show the expected inverse association with pro-inflammatory factors, whereas TGF-β1 was correlated with IL-33, IL-1β, and IL-4. Furthermore, the pro-inflammatory chemokines CCL-2 and CXCL-10 were negatively correlated with the anti-inflammatory cytokines IL-4 and IL-33. In a subphenotype analysis, levels of IL-1β and IL-4 (cluster 4) were higher in ST than in MT (Supplementary Fig. S3A), and IL-33 levels increased with the severity of toxoplasmosis (Supplementary Fig. S3B). Only the TGF-β1 level was significantly higher in SM and in MM (Supplementary Fig. S3C). These data revealed distinct cytokine profiles associated with the various protozoan infections; these profiles might correspond to different immune response mechanisms and could potentially serve as specific biomarkers of disease severity and the type of infection.

### Biochemical factors and inflammatory processes that identify patients with malaria, toxoplasmosis and leishmaniasis co-infections

**Fig. 8:**
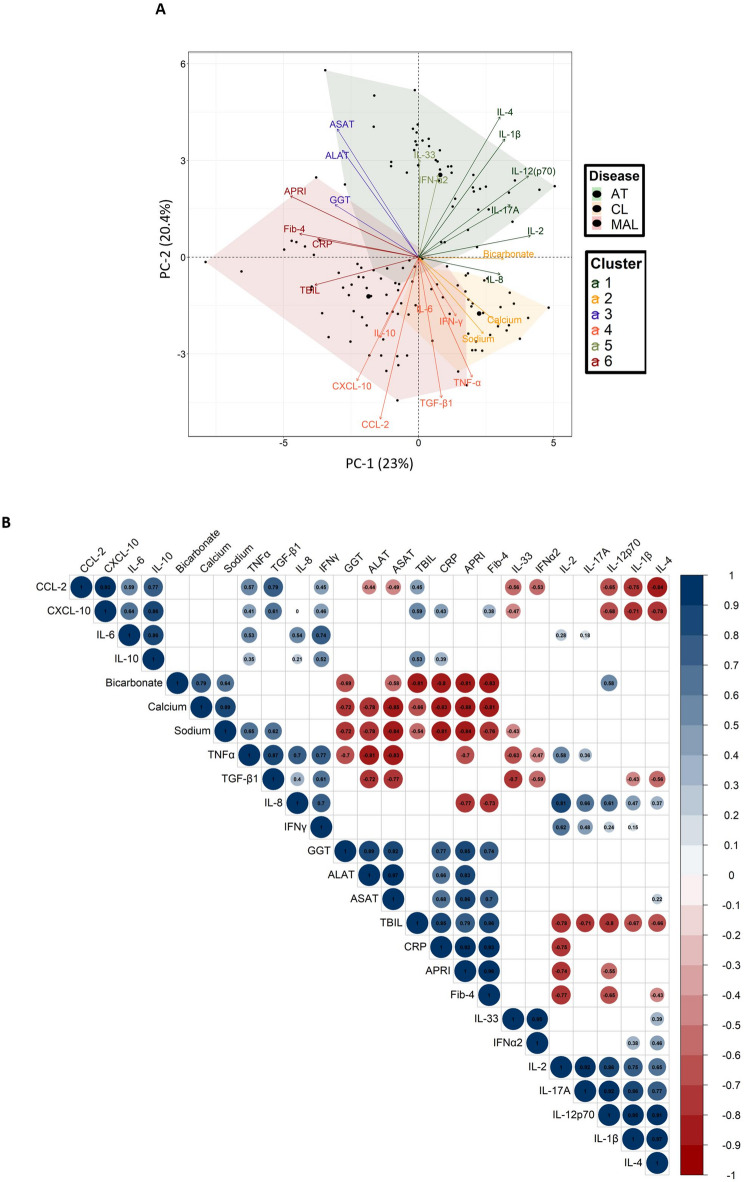
Specific profiles of factors discriminate between disease groups. **A** Biplot of a principal component analysis of individuals for the first two principal components (explaining 43.4% of the total variance). Patients with missing values < 6 were excluded from the analysis. Variables with missing values were imputed with the mean value for the variable. Six clusters were determined using a *k-*means algorithm, in order to assess the relationships between biochemical and inflammatory factors. **B** Spearman’s correlations. Only the correlation with *p* < 0.01 are shown. The size of the spot is proportional to the strength of the correlation, and the colour of the spot corresponds to the direction (blue for a positive correlation and red for a negative correlation). The value of the correlation coefficient is noted in the spot, and the scale is given on the right.

We used a PCA to evaluate the relatedness of 31 biochemical factors and cytokine/chemokine responses among the MAL, AT and CL groups. Our analysis of 130 patients revealed six distinct clusters (Fig. 8A). Cluster 1 consisted of IL-1β, IL-2, IL-4, IL-8, IL-12(p70) and IL-17 A. Cluster 2 included plasma bicarbonate, sodium, and calcium. Cluster 3 featured GGT, ALAT, and ASAT. Cluster 4 was composed of IL-6, IL-10, CXCL-10, CCL-2, IFN-γ, TGF-β1 and TNF-α. Cluster 5 comprised IL-33 and IFN-α2. Cluster 6 included TBIL, CRP, FIB-4 and APRI. The AT group was characterized by clusters 1, 3 and 5. The MAL group was characterized by clusters 4 and 6. Lastly, the CL group was characterized by a mix of clusters 1, 2, and 4. With the exception of TBIL, CRP, and FIB-4, all the biochemical factors were negatively correlated with the cytokine levels except sodium with TGF-β1 and TNF-α (Fig. 8B). A subsequent PCA aimed to differentiate multi-infected from mono-infected patients across the cohorts (not shown) failed to reveal clear distinction between individuals with previous parasitic infection and those with current infected patients.

**Fig. 9:**
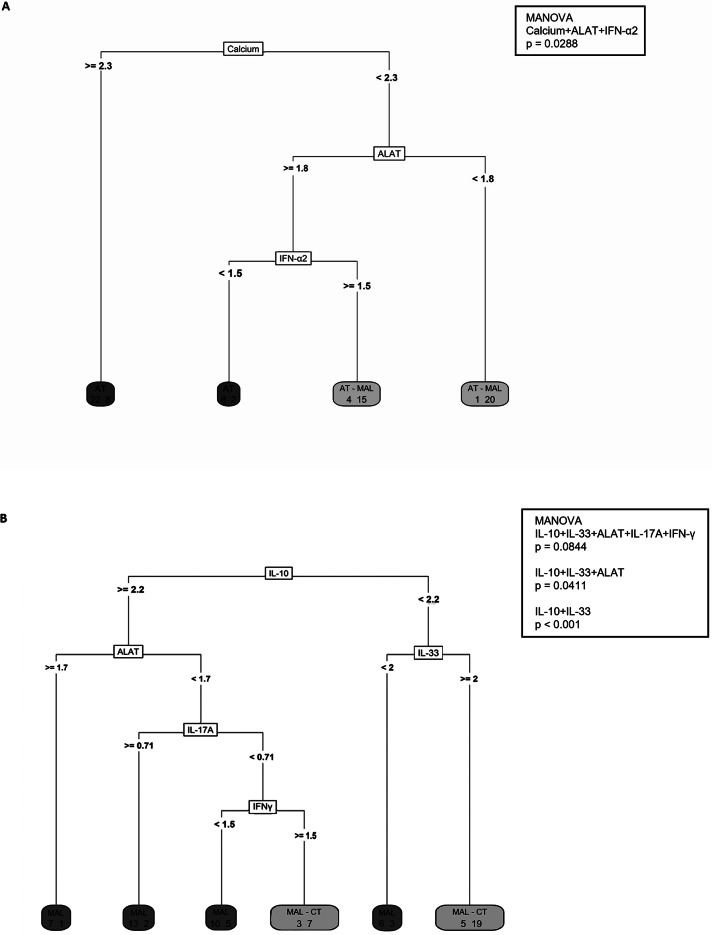
Classification tree models. The recursive partitioning and regression trees were based on previously detected discriminant variables. A minimum of 14 patients was required to split the nodes, and the minimum number of patients in a terminal node was five. Patients with toxoplasmosis (*n* = 78) were correctly classified in 85% and 78% of cases for AT and AT - MAL, respectively (**A**) and patients with malaria (*n* = 81) were correctly classified in 81% and 70% of cases for MAL and MAL - CT, respectively (**B**). A multivariate analysis of variance with Pillai’s trace method was used to assess the statistical significance of the association between the set of factors and the group.

A classification tree analysis identified critical determinants of specific disease phenotypes (Fig. 9). The classification tree accurately classified 85% of toxoplasmosis cases in the AT group (*n* = 28) and 78% of cases in the AT - MAL co-infected group (*n* = 35). Furthermore, the classification tree successfully identified 82% of malaria cases in the MAL group (*n* = 36) and 70% in the MAL - AT group (*n* = 26). Distinguishing features in the AT - MAL group (relative to the AT group) included lower calcium and ALAT levels and a higher IFN-α2 level (Fig. 9A). Relative to the MAL group, the MAL - CT group had lower IL-10, ALAT and IL-17 A, levels and higher IL-33 and IFN-γ (Fig. 9B). A multivariate analysis of variance revealed significant differences for IL-10, IL-33 (*p* = 1.21 × 10^-11^ for both) and ALAT (*p* = 0.0411). No distinct profiles emerged for the CL subgroups, which were segregated by individual cytokines and chemokines only.

**Fig. 10:**
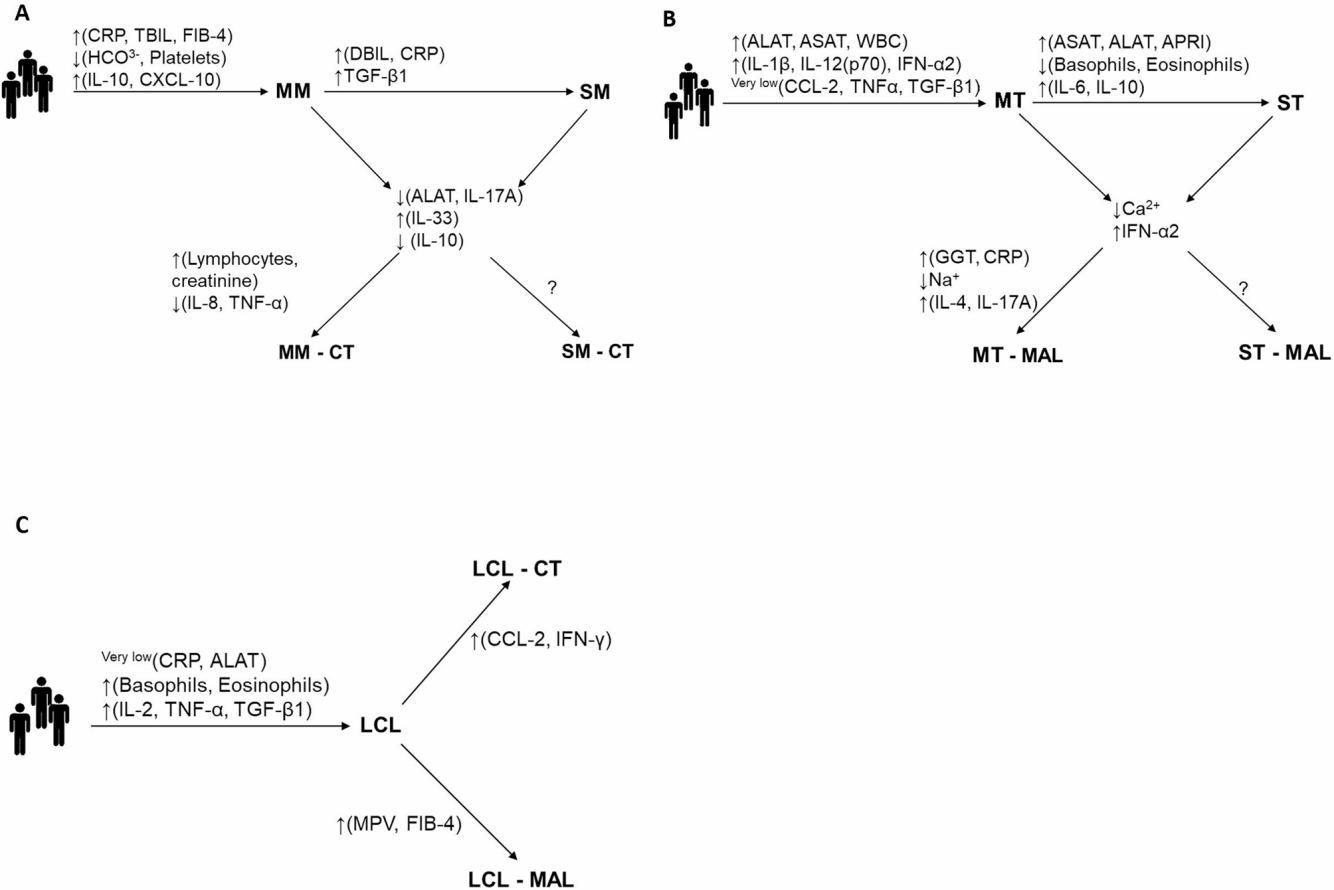
A model of disease outcome, according to the multi-infection. The models were built from the pooled results of the bivariate and multivariate analyses and the results of the tree regression models. The first set of factors are those that distinguished between the three diseases. **A** The model for MAL patients. **B** the model for AT patients. **C** the model for patients with localized CL. The groups were formed independently of a history of infection with a third parasite.

Bivariate analyses were used to identify factors that highlighted clinical subgroups of sequentially co-infected individuals (see Fig. S4, S5, and S6) and then develop a model that discriminated between disease groups and subphenotypes (Fig. 10). Compared with the MM group, the MM - CT subgroup showed higher creatinine levels, lower Modification of Diet in Renal Disease scores, higher lymphocyte counts, and lower levels of IL-8, IL-17 A, and TNF-α (Fig. 10A). The MT group exhibited higher GGT and CRP levels and lower plasma sodium than the MT - MAL co-infected group; the latter displayed elevated IL-4 and IL-17 A levels (Fig. 10B). No differences between the CL subgroups were observed. Further analysis of the impact of sequential co-infection with *T. gondii* or with *Plasmodium* on the severity of leishmaniasis revealed a higher degree of fibrosis (i.e. an elevated FIB-4 index) and a higher MPV in the LCL - MAL group than in the LCL group, and higher CCL-2 and IFN-γ levels in the LCL - CT group than in the LCL group (Fig. 10C). These results highlight the complexity of the interactions between biochemical factors and inflammatory processes in patients with multiple protozoan infections and thus provide valuable insights into the intricate pathophysiology of these diseases.

## Discussion

While infectious diseases worldwide are well documented, data on protozoan co-infections are relatively scarce^36–43^. Although Western Blots and specific neutralization assays for each protozoan would have further to strengthen the results, our study revealed a high prevalence of protozoan co-infections (specifically involving *T. gondii*, *Plasmodium* spp., and *Leishmania* spp.) among individuals in French Guiana. In our study of 253 patients, 2.4% had concurrent infections (AT or CL with MAL). Serological tests showed that approximately 60% of participants had been exposed to at least two of the parasites studied. Notably, over half of the participants with MAL or CL had been infected with *T. gondii*, which suggests that they were suffering from CT. Furthermore, a significant proportion of these patients displayed antibody responses to *Plasmodium* - underscoring the high prevalence of co-infections in the MAL population. However, only patients with available blood samples were included in the study, and so this selection criterion probably led to underestimation of the true prevalence of concurrent, acute infections. For instance, public health data in the literature indicate a prevalence of CL of 2.4% to 8.3% among illegal gold miners with MAL^14,44^. A particularly intriguing case featured a patient with AT and MAL who exhibited no overt symptoms of toxoplasmosis, despite serological evidence of recent *T. gondii* seroconversion and concurrent *P. vivax* parasitaemia. Even though this was a single case report, it suggested that co-infections mitigate the effect of disease symptoms. These findings underscore the critical importance of considering co-infections in clinical diagnoses and patient management strategies and highlight the complex interactions that can influence disease presentation and treatment outcomes. In addition, there were more cases of AT - MAL infection in the dry season than in other seasons. This could suggest that the low prevalence of concomitant infections found in this study could also be linked to a limited overlap of transmission seasons for these parasites in the region. Moreover, our study had a number of limitations, such as inability to fully determine the extent of asymptomatic carriage in patients with a positive serological assay for *Plasmodium* spp. or *T. gondii.* Asymptomatic carriage of *Plasmodium* spp. is common^45–47^ and often associated with biological abnormalities, such as anaemia and thrombocytopenia^48^. Asymptomatic carriage has often been observed in patients with a high titre of IgGs against *Plasmodium* and gsG6-P1 (a marker of recent exposure to *Anopheles* bites)^49,50^. Interestingly, a quarter of the patients in the AT group were negative for *Plasmodum* spp. but displayed much the same total anti-*Plasmodium* Ig levels as MAL patients; this suggests asymptomatic, submicroscopic *Plasmodium* carriage and so, underestimated concurrent infection. In our data, patients with sequential infections also tended to have a lower *Plasmodium* parasite load and a lower number of LCL lesions, which could have an impact on diagnosis. More generally, multi-infected patients showed a slight decrease in symptom severity, and the high seroprevalence of multiple infections with *T. gondii*, *Plasmodium* spp. and/or *Leishmania* spp. could favour mild or even asymptomatic infections in the region. Nevertheless, a prospective study on a larger cohort would be necessary to study this with an appropriate depth. Our analysis encompassed a broad array of factors evaluated in routine patient care and therefore yielded some valuable insights. Nonetheless, specific aspects of the diagnosis and management of each disease constrained the scope of our multivariate and regression analyses. Despite these limitations, we identified distinct clusters of factors and cytokine/chemokine signatures that differentiated between AT patients, MAL patients and (to a lesser extent) CL patients. For the latter, no difference was observed between LCL and DL patients and they were analyzed at the same time. Notably, levels of distinct biochemical analytes (such as the liver damage markers TBIL, ALAT, ASAT, and GGT) were significantly lower in CL patients; hence, these analytes might be biomarkers for AT or MAL^51^. This hypothesis is supported by the hepatic impairments (hepatomegaly, jaundice, and cytolysis) observed in the AT and MAL groups. Furthermore, a significantly lower platelet count in the MAL group highlighted a distinct, platelet-mediated, anti-parasite response, which further underscored the complex interplay between biochemical responses to protozoan infections^52^.

However, our detailed characterization of cytokine and chemokine profiles offered the most intriguing insights and showed that biomarkers can differentiate between infection groups. One of our most striking findings was the segregation of distinct cytokine/chemokine clusters for the MAL, AT, and CL groups. The MAL group was primarily identified by elevated plasma levels of the cytokines IL-6, IL-10, CXCL-10, CCL-2, IFN-γ, TGF-β1, and TNF-α. This profile underscores the intense immune response triggered by *Plasmodium* infection, which was predominantly mediated by IFN-γ and modulated by IL-10. In *P. vivax* malaria, the immune response is characterized by elevated plasma levels of IL-6, IL-17, CCL-2, and CXCL-10, and the severity of anaemia is linked to high levels of IFN-γ and TNF-α. In contrast, patients with *P. falciparum* MAL in India displayed higher levels of IFN-γ, IL-2, IL-6 and IL-12, and disease severity was associated with the high levels of TGF-β, TNF-α, CXCL-10, IL-1β, IL-10^53,54^.

The cluster characterizing the AT group included different chemokines and cytokines: IL-1β, IL-2, IL-4, IL-8, IL-12(p70), and IL-17 A. This finding was consistent with previous work^55^ and highlight the critical role of early IL-12 production in *T. gondii* resistance, with the proliferation of IFN-γ-producing cells^56^. The balance between pro- and anti-inflammatory responses is mediated by IL-12/IFN-γ and IL-4/IL-33 and characterizes the immune response to *T. gondii*^57,58^. These findings are particularly relevant because they suggest that exposure to *Plasmodium* enhances the anti-*T. gondii* innate response, possibly reducing parasitaemia and inducing a more potent specific T_H_2 response (*via* IL-4 and IL-33).

The CL group’s response involved clusters of cytokines and chemokines typically associated with both MAL and AT groups, and did not differ between clinical subgroups LCL and DL. This blend of T_H_1, T_H_2, and T_H_17 responses indicates that the immune response in CL is complex. Disease progression is reflected by a transition from a T_H_2 response (IL-4 and IL-10) to a T_H_1 response (TNF-α and IFN-γ), together with high levels of IL-1β, IL-6, and IL-17^59–62^. The presence of high levels of TGF-β1 in our patients might explain (at least in part) the prolonged recovery from CL and suggests that MPV, TGF-β1, and TNF-α are potential biomarkers for this disease^63–65^.

The impact of *T. gondii*, *Plasmodium*, and *Leishmania* co-infections on the immunophysiopathology of each individual disease is profound - even in the absence of severe clinical phenotypes. Interestingly, our results and some literature data indicate that co-infection with *T. gondii* protects against the liver dysfunction typically associated with MAL^54,66^. Furthermore, our observations of impaired renal function and higher lymphocyte counts in co-infected MAL patients open up new avenues for investigating the immunological mechanisms underlying co-infections^67–71^.

We used a recursive partitioning approach to assess the most significant variables for distinguishing subgroups of multi-infected individuals. However, no cross-validation was possible due to the heterogeneity of the database, and so we can’t exclude overfitting and loss of external validity explaining potential divergences from more comprehensive data. Still, we comforted the significance of respective sets of variables by parallel MANOVA. A particularly noteworthy finding was the modulation of IL-17 in response to IL-33, which promotes the recruitment of IFN-γ-producing lymphocytes^69,70^. This modulation suggests that a decrease in IL-10 in MAL patients co-infected with *T. gondii* is linked to the decrease in the T_H_17 response^68^. Moreover, our data indicate that co-exposure to MAL exacerbates the severity of AT in patients. This might be due to elevated levels of pro-inflammatory cytokines and might constitute a side effect of the host’s increased resistance to *T. gondii* infection^72^. The distinct immune responses in the AT - MAL group (characterized by low plasma calcium and ALAT levels and a high IFN-α2 level) highlight the complexity of the balance between T_H_1 and T_H_2 responses during a *T. gondii* infection. Finally, through our models, we highlight signatures that also distinguish mild from severe patients and may therefore help to understand how previous infections affect the pathophysiology of a subsequent disease. Yet, the precise impact of previous infection in the SM - CT and ST - MAL groups remains unknown due to the lack of significant data, but suggests a milder profile. However, even though we observed specific interactions and marginal influences of pre-existing infections and co-infections, the overall responses were predominantly determined by the primary infectious agents.

In conclusion, the results of the present study (i) suggest that protozoan co-infections are highly prevalent in endemic regions such as French Guiana, (ii) identified key biomarkers of these co-infections, and (iii) highlighted the interest of an integrated approach that combines biochemical, cellular, and cytokine/chemokine profiling for the diagnosis, management, and comprehension of these infections. Our findings pave the way for further research (including longitudinal studies) of the co-infections’ impact on disease progression, treatment responses, and parasite transmission dynamics in endemic settings.

## Materials and methods

### Study design and population

The present study was carried out at the Cayenne hospital Center (Cayenne, French Guiana, France) and a number of health centres in the remote areas of French Guiana. All methods were carried out in accordance with relevant guidelines and regulations. We retrospectively studied patients suffering from acute toxoplasmosis (AT), MAL and/or CL between 2012 and 2022, regardless of geographical location or season. The cohort was extended with endemic controls (EC) from another study^73^. We included adult patients (18- to 77-year-old) with a blood sample available. Pregnant women and people immunocompromised by HIV or chemotherapy were excluded. Blood was collected during admission and stored at -80 °C. The concomitant or sequential infection with *T. gondii*, *Plasmodium* spp. or *Leishmania* spp. were assessed secondarily as described below. Parameters were collected during hospitalisation and completed with cytokine and serological assays for the research. Patients with other active infections or outlier data were excluded from the study (Fig. 1).

### Infection definitions and endemic controls

Single infections and co-infections with *T. gondii*, *Plasmodium* spp. or *Leishmania* spp. were characterized as follows. AT patients were diagnosed as described previously^30^. Briefly, a confirmed case of AT was defined by a serological diagnosis of acute infection (i.e. a *Toxoplasma* profile with a significant rise in IgG serological titres between two consecutive toxoplasmosis serologies and anti-*Toxoplasma* IgM index ≥ 4), regardless of whether or not a PCR assay had detected *T. gondii* DNA in a biological fluid. A confirmed case of AT was also defined as a single positive serological titre point and a positive PCR assay. Depending on the severity of the infection, the patients with AT were classified as having mild toxoplasmosis (MT, i.e. mild symptoms and uncomplicated prolonged fever) or severe toxoplasmosis (ST, requiring admission to hospital or even an intensive care unit). Severe complications included respiratory distress with or not insufficiency, hepatic cytolysis (defined as aspartate aminotransferase (ASAT) and alanine aminotransferase (ALAT) levels more than twice the upper reference value), heart disease (troponin level > 0.014 µg/L), ophthalmological abnormalities, and multi-organ failure. MAL group was defined using a rapid diagnostic test (Bioline™ MALARIA Ag P.f/Pan, Abbott, USA), coupled with identification of the species by microscopic observation of a thick blood smear. A multiplex PCR assay^74^ was used to confirm the identity of the plasmodial species and detect *P. vivax*-*P. falciparum* co-infections. All infected patients were classified as having uncomplicated or severe malaria, according to the 2012 World Health Organization (WHO) guidelines (for *P. falciparum*) or modified guidelines (for *P. vivax*)^75,76^. Next, two groups were defined for each of *Plasmodium* species: (i) mild malaria (MM) with fever but no severity criteria ; and (ii) severe malaria (SM) with at least one severity criterion, such as a haemoglobin level < 7 g/dL, acidosis (plasma HCO_3_^-^ < 15 mmol/L or venous lactate > 5 mmol/L), acute renal injury (creatinine > 3 mg/dL or urea > 20 mmol/L), or jaundice (bilirubin ≥ 3 mg/dL)^77^. In our study, the main disease criteria were thrombopenia and anaemia. In the subgroup infected with *P. vivax*, most had MM. Patients infected with *P. falciparum* had returned from a trip to sub-Saharan Africa. CL was diagnosed after a clinical examination and detection of an active, *Leishmania*-containing lesion. The infection was confirmed by May-Grünwald Giemsa-stained skin smears, a positive culture, and/or the detection of the parasite by PCR restriction fragment length polymorphism at the French National Reference Centre for Leishmaniases^78^. Patients with an *L. guyanensis* infection were classified into three groups, as described previously^59^: (i) patients with localized CL (LCL) had 1 to 15 ulcerations on one or several body sites; (ii) patients had disseminated leishmaniasis with the presence of more than 10 polymorphic cutaneous lesions on at least two non-contiguous body segments; (iii) patient had mucocutaneous leishmaniasis with nasal ulceration. Patients infected with *L. braziliensis* or non-identified *Leishmania* species were classified as having LCL. Most CL patients had one to five lesions; these were mainly ulcerations. Furthermore, plasma samples from 19 endemic control (EC) patients were obtained from Dr Maylis Douine (Antilles-Guyane Clinical Investigation Centre), in compliance with the French legislation. The ECs had a negative blood smear/thick drop test for *Plasmodium*, no *Leishmania* lesions, and no diagnosis of toxoplasmosis. The EC plasma samples were used to quantify cytokine and chemokine levels.

Patients with a sequential co-infection were defined as having AT, MAL or CL and a history of at least one of the other two diseases. The seroprevalence of these infections was assessed using an ELISA or a chemiluminescent microparticle immunoassay (CMIA). Patients positive for *T. gondii*-specific IgG but negative *T. gondii*-specific IgM were considered to have chronic toxoplasmosis (CT); they were then classified into five sequential co-infection groups (AT - MAL, MAL - CT, CL - CT, CL - MAL and CL - CT - MAL). Patients infected simultaneously with AT, MAL and/or CL constituted the two groups of concomitant co-infections (AT + MAL and MAL + CL). Patients exposed solely to AT, MAL or CL constituted the AT, MAL and CL groups, respectively (Fig. 1).

### Ethical considerations

This study falls within the scope of Research that does not involve the human person and falls under the “Reference Methodology” (MR004) for which the Cayenne hospital Center has signed a compliance undertaking dated 21/12/2021. All experimental protocol were approved by the Cayenne hospital Center’s ethical committee. The sample library was registered with the French Ministry of Education and Research (references: DC-2013-2022 and DC-2021-4666). A privacy impact assessment has been carried out, and a summary of the study has been published on the Health-Data-Hub website (https://www.health-data-hub.fr/) under number F20210817194527. The study was conducted according to the French guidelines on retrospective studies (the MR004 reference methodology). The legal basis for data processing is the public interest mission. All the data used are taken from patients’ medical records, and obtained in the course of routine care. The database has been pseudo-anonymized. In compliance with French law, the CNIL and the European Union’s General Data Protection Regulation, which are among the most restrictive in the world, individual and collective information has been provided to patients. No additional formalities are required in France. Furthermore, the Cayenne Hospital Center does not have a local ethics committee dedicated to research projects.

### Information consent

Informed consent was obtained from all subjects and/or their legal guardians. An information note was sent to the patient’s postal address. In the absence of any expression of opposition from the patient within one (1) month of the date of dispatch of the letter, it was considered that the patient had not objected to participating in the study. If the non-opposition letter was returned with the note “not living at the address indicated”, or if we had not received a reply within 1 month, the patient was included in the study “by default”. If the patient was deceased, and had not objected to the processing of his/her data during his/her lifetime, he/she was also included “by default”. If the patient objected to the processing of his/her data, he/she made this known using the objection form attached to the information note, by e-mail or by any other means at his/her disposal to the project leader or to the dedicated data protection officer.

### Blood collection

Venous blood samples from all included patients were collected in sterile vacutainers with EDTA before treatment with sulfamethoxazole-trimethoprim for AT (depending on the context of the outbreak), pentamidine for CL, and quinine, quinine derivatives or artemether-lumefantrine for MAL. Blood samples were collected from some patients after the start of preventive treatment. Plasma was obtained by centrifuging blood samples at 1500 *g* for 15 min. The plasma samples were stored at − 80 °C until use.

### Culture of *L. guyanensis* promastigotes and preparation of crude antigens

*L. guyanensis* isolated from skin biopsies was cultured in RPMI 1640 medium (Gibco, Paisley, UK) containing L-glutamine and 25 mM HEPES supplemented with 20% heat-inactivated FBS, (Gibco), 50 IU/mL penicillin, 0.05 mg/mL streptomycin and nonessential amino acids (Gibco). The cultures were maintained until numerous, large clumps of parasites had formed. Next, the culture was centrifuged at 300 *g* for 5 min at room temperature. The supernatant was discarded, and the promastigote pellet was resuspended in the same medium without foetal bovine serum. After two days, the parasites were pelleted by centrifugation at 1500g for 15 min at 4 °C and resuspended in cold PBS three times. The parasite pellet was then resuspended in RIPA lysis buffer (Interchim, Montluçon, France) containing a cocktail protease inhibitor (catalogue number #4693159001, Roche, Boulogne-Billancourt, France). For lysis and protein extraction, 10^9^ parasites were resuspended in 1 mL of RIPA lysis buffer. After a 10-minute incubation at room temperature, the cell lysate was frozen/thawed five times in liquid nitrogen. The extracts were then assayed for protein in a BCA assay (catalogue number #23227, Pierce, France), adjusted to 1 mg/mL, and stored at – 20 °C until use.

### Quantification of parasite-specific immunoglobulins G and M

The plasma concentrations of IgM and IgG against various parasite-specific antigens were measured for each patient using a CMIA or an ELISA. IgG and IgM specific for *T. gondii* SAG-1 and GRA-8 antigens were assayed using the Toxo IgG and Toxo IgM kits for Architect i2000 (Abbott Diagnostics, Abbott Park, IL, USA). The sample was considered to be positive for *T. gondii* when the IgG level was ≥ 3 IU/mL and/or the IgM level was ≥ 0.6 (index). Total levels of IgG and IgM against to *Plasmodium* MSP-1 were measured using the Malaria EIA kit (catalogue number #72526, BioRad, France), according to the manufacturer’s instructions. Positivity was defined as an O.D. greater than the cut-off value. For the measurement of specific anti-*L. guyanensis* antibodies, the ELISA plates were coated with 5 µg/mL of crude protein at 4 °C overnight. Next, wells were blocked with 1% gelatine in PBS for 90 min, and the plasma was incubated for 90 min at 37 °C at a dilution of 1/200. The plates were then incubated at 37 °C with a human anti-IgG (1/10000) coupled to peroxidase (Sigma, France). The presence of specific antibodies was revealed using 3,3’,5,5’-tetramethylbenzidine (TMB) substrate. The enzyme reaction was stopped with dilute sulphuric acid, and the optical density was measured at 450 nm. A plasma sample from a leishmaniasis-negative patient was used as a negative control, and a pool of plasma from positive acute-phase patients was used as a positive control.

### Database collection

The data collected include: (i) sociodemographic and epidemiological information (the biotope of residence, meat-borne risk factors, living and working in the forestry, etc.); (ii) biochemical variables (blood counts, renal and liver function assays, electrolyte levels, coagulation profile, and disease biomarkers), and other infectious agents (the serological status for HIV and viral hepatitis, tuberculosis, Q fever, leptospirosis, or infection with *Histoplasma capsulatum*, helminths or other protozoans); (iii) the reasons for hospital admission, the results of clinical examinations (disease outcomes and comorbidities such as toxicological status, metabolic disorders, and hypertension) and medications; and (iv) any other relevant information in the patient’s medical records (Supplementary Table 1).

### Plasma cytokine assays

Plasma concentrations of 15 cytokines involved in host-parasite responses i.e. IL-1β, IL-2, IL-4, IL-6, IL-8, IL-10, IL-12(p70), IL-17 A, IFN-γ, chemokine (C-C motif) (CCL)-2, C-X-C motif chemokine ligand (CXCL)-10, TNF-α, and transforming growth factor (TGF)-β1 (catalogue number #740930, Human Essential Immune Response Panel 13-plex, BioLegend Paris, France) as well as IL-33 and IFN-α2 (catalogue number #741378, Human Cytokine Panel 2, BioLegend) were measured using a BD LSRFortessa^™^ flow cytometer (BD Biosciences, USA) equipped with a high-throughput sampler, according to the manufacturer’s instructions. Data were analyzed using the LEGENDplex^™^ Data Analysis Software. Briefly, the samples were prepared in V-bottom plates and incubated overnight with shaking at around 450 rpm. The beads were washed once after each incubation and analyzed on the flow cytometer. Three hundred events per analyte were recorded, and concentrations were compared with a standard curve to determine the relative concentration. The limits of detection were as follows: IL-1β, 18.3 pg/mL; IL-2, 0.4 pg/mL; IL-4, 5.4 pg/mL; IL-6, 5.2 pg/mL; IL-8, 10.3 pg/mL; IL-10, 1.1 pg/mL; IL-12(p70), 3.3 pg/mL; IL-17 A, 1.1 pg/mL; IFN-γ, 6.3 pg/mL; CCL-2, 6.1 pg/mL; CXCL-10, 7.8 pg/mL; TNF-α, 2.1 pg/mL; TGF-β1, 27.0 pg/mL; IL-33, 65.3 pg/mL; IFN-α2, 6.02 pg/mL.

### Statistical analysis

All statistical analyses were performed using R software (version 4.2.2); (http://www.R-project.org). The normality of the data was checked using the Shapiro-Wilk test, and the data were log-transformed when appropriate. An initial descriptive analysis of the study population was conducted using the R DescTool package (version 0.99.5). Multinomial logistic regression was used for categorical variables. The Venn diagram of the seroprevalence to *T. gondii*,* Plasmodium* spp. and *Leishmania* spp. in the study population as a whole was generated using the R VennDiagram package (version 1.7.3). Bivariate analyses using the R ggpubr package (version 0.6.0) were used to assess differences between the single-infection groups and the sequential multi-infection groups. Missing values were denoted as “not available”. For multiple comparisons, the Kruskal–Wallis test was followed by the Benjamini-Hochberg (BH) correction. Next, a post-hoc Conover’s test with the BH correction was used for pairwise comparisons of the groups that were significant in the Kruskal-Wallis test. A principal component analysis (PCA) was performed and visualized using the R packages FactoMineR (version 2.8) and factoextra (version 1.0.7), respectively. Missing values were imputed with the mean. The type of ellipse assumed a multivariate t-distribution with a 95% confidence interval (CI), and the variables used in the PCA were coloured using a *k*-means clustering algorithm. Correlations were estimated from linear regressions, using the R Hmisc (v5.1-1) package. Following the selection of relevant variables in the segregation of the three disease groups, a classification tree model was applied to determine the most impactful variables that discriminated the patient subgroups^79^. The classification tree was built using the R packages rpart (version 4.1.19) and rpart.plot (version 3.1.1). The entire sample was used to build the model, with a process selecting the most relevant variable and threshold to split the data into two groups at each step. The recursive splitting continues until the minimum size into the terminal nodes is met or until no improvement can be made. Confusion matrix was used to assess the accuracy of the prediction.

## Supplementary Information

Below is the link to the electronic supplementary material.

Supplementary Material 1

Supplementary Material 2

## Data Availability

The datasets used and/or analysed during the current study are available from the corresponding author on reasonable request.

## References

[1] Kamuyu, G. et al. Exposure to multiple parasites is associated with the prevalence of active convulsive epilepsy in sub-Saharan Africa. *PLoS Negl. Trop. Dis.* **8**, e2908 (2014). 24875312 10.1371/journal.pntd.0002908 PMC4038481

[2] Temporão, A. et al. Excreted Trypanosoma brucei proteins inhibit Plasmodium hepatic infection. *PLoS Negl. Trop. Dis.* **15**, e0009912 (2021). 34714824 10.1371/journal.pntd.0009912 PMC8580256

[3] Menezes, R. A. D. O. et al. Enteroparasite and vivax malaria co-infection on the Brazil-French Guiana border: Epidemiological, haematological and immunological aspects. *PLoS ONE*. **13**, e0189958 (2018). 29293589 10.1371/journal.pone.0189958 PMC5749708

[4] Onkoba, N. W., Chimbari, M. J. & Mukaratirwa, S. Malaria endemicity and co-infection with tissue-dwelling parasites in Sub-Saharan Africa: A review. *Infect. Dis. Poverty*. **4**, 35 (2015). 26377900 10.1186/s40249-015-0070-0 PMC4571070

[5] Costa, A. S. L., Santos, V. C., Amorim, P. C. V., Santos, J. E. & Pinto, A. Y. D. N. Infecção tripla por Trypanosoma cruzi, Plasmodium vivax e P. falciparum: relato de caso. *J. Bras. Patol. Med. Lab.* **48**, 421–425 (2012).

[6] Rezende-Oliveira, K. et al. Effects of meglumine antimoniate treatment on cytokine production in a patient with mucosal leishmaniasis and chagas diseases co-infection. *TropicalMed* **5**, 69 (2020). 10.3390/tropicalmed5020069 PMC7345053 32370270

[7] Barrett, M. P., Kyle, D. E., Sibley, L. D., Radke, J. B. & Tarleton, R. L. Protozoan persister-like cells and drug treatment failure. *Nat. Rev. Microbiol.* **17**, 607–620 (2019). 31444481 10.1038/s41579-019-0238-x PMC7024564

[8] Griffiths, E. C., Pedersen, A. B., Fenton, A. & Petchey, O. L. The nature and consequences of coinfection in humans. *J. Infect.* **63**, 200–206 (2011). 21704071 10.1016/j.jinf.2011.06.005 PMC3430964

[9] Rigaud, T., Perrot-Minnot, M. J. & Brown, M. J. F. Parasite and host assemblages: Embracing the reality will improve our knowledge of parasite transmission and virulence. *Proc. R Soc. B*. **277**, 3693–3702 (2010). 20667874 10.1098/rspb.2010.1163 PMC2992712

[10] De Thoisy, B. et al. Ecology, evolution, and epidemiology of zoonotic and vector-borne infectious diseases in French Guiana: Transdisciplinarity does matter to tackle new emerging threats. *Infect. Genet. Evol.* **93**, 104916 (2021). 34004361 10.1016/j.meegid.2021.104916

[11] Confalonieri, U. E. C., Margonari, C. & Quintão, A. F. Environmental change and the dynamics of parasitic diseases in the Amazon. *Acta Trop.* 33–41. 10.1016/j.actatropica.2013.09.013 (2013). 10.1016/j.actatropica.2013.09.013 24056199

[12] World Health Organization, C. *World Malaria Report 2023* (World Health Organization, 2023).

[13] Pan American Health Organization. Report on the Situation of Malaria in the Americas – 2017. 1–9. (2017).

[14] Douine, M. et al. Illegal gold miners in French Guiana: A neglected population with poor health. *BMC Public. Health*. **18**, 23 (2018). 10.1186/s12889-017-4557-4 PMC5513330 28716015

[15] Musset, L. et al. Emergence of plasmodium vivax resistance to chloroquine in French Guiana. *Antimicrob. Agents Chemother.* **63**, e02116–e02118 (2019). 31481442 10.1128/AAC.02116-18 PMC6811453

[16] Rishikesh, K. & Saravu, K. Primaquine treatment and relapse in *Plasmodium vivax* malaria. *Pathogens Global Health*. **110**, 1–8 (2016). 27077309 10.1080/20477724.2015.1133033 PMC4870028

[17] Price, R. N. et al. Global extent of chloroquine-resistant Plasmodium vivax: A systematic review and meta-analysis. *Lancet. Infect. Dis*. **14**, 982–991 (2014). 25213732 10.1016/S1473-3099(14)70855-2 PMC4178238

[18] Leonard, C. M. et al. Missed Plasmodium falciparum and Plasmodium vivax mixed infections in Ethiopia threaten malaria elimination. *Am. J. Trop. Med. Hyg.* **106**, 667–670 (2022). 10.4269/ajtmh.21-0796 PMC8832938 34847530

[19] Faure, E. Malarial pathocoenosis: Beneficial and deleterious interactions between malaria and other human diseases. *Front Physiol* **5**, (2014). 10.3389/fphys.2014.00441 PMC4240042 25484866

[20] Burza, S., Croft, S. L., Boelaert, M. & Leishmaniasis *Lancet* **392**, 951–970 (2018). 30126638 10.1016/S0140-6736(18)31204-2

[21] Pasquier, G. et al. Leishmaniasis epidemiology in endemic areas of metropolitan France and its overseas territories from 1998 to 2020. *PLoS Negl. Trop. Dis.* **16**, e0010745 (2022). 36206322 10.1371/journal.pntd.0010745 PMC9624409

[22] Jagadesh, S. et al. Spatial variations in Leishmaniasis: A biogeographic approach to mapping the distribution of Leishmania species. *One Health*. **13**, 100307 (2021). 34430698 10.1016/j.onehlt.2021.100307 PMC8368019

[23] Ponte-Sucre, A. et al. Drug resistance and treatment failure in leishmaniasis: A 21st century challenge. *PLoS Negl. Trop. Dis.* **11**, e0006052 (2017). 29240765 10.1371/journal.pntd.0006052 PMC5730103

[24] Gangneux, J. P. et al. Recurrent American Cutaneous Leishmaniasis. *Emerg. Infect. Dis.* **13**, 1436 (2007). 18252137 10.3201/eid1309.061446 PMC2857276

[25] Dedet’, J. P., Pradinaud, R. & Gay’, F. Aspects of human cutaneous leishmaniasis in French. 10.1016/0035-9203(89)90375-1 2617622

[26] Conceição-Silva, F., Leite-Silva, J. & Morgado, F. N. The binomial parasite-host immunity in the healing process and in reactivation of human tegumentary leishmaniasis. *Front. Microbiol.* **9**, 1308 (2018). 29971054 10.3389/fmicb.2018.01308 PMC6018218

[27] Robert-Gangneux, F. & Dardé, M. L. Epidemiology of and diagnostic strategies for toxoplasmosis. *Clin. Microbiol. Rev.* **25**, 264–296 (2012). 22491772 10.1128/CMR.05013-11 PMC3346298

[28] Dardé, M. L., Villena, I., Pinon, J. M. & Beguinot, I. Severe toxoplasmosis caused by a *Toxoplasma gondii* strain with a new isoenzyme type acquired in French Guyana. *J. Clin. Microbiol.* **36**, 324–324 (1998). 9431981 10.1128/jcm.36.1.324-324.1998 PMC124868

[29] Carme, B., Demar, M., Ajzenberg, D. & Dardé, M. L. Severe acquired toxoplasmosis caused by wild cycle of *Toxoplasma gondii*, French Guiana. *Emerg. Infect. Dis.* **15**, 656–658 (2009). 19331765 10.3201/eid1504.081306 PMC2671434

[30] Demar, M. et al. Acute toxoplasmoses in immunocompetent patients hospitalized in an intensive care unit in French Guiana. *Clin. Microbiol. Infect.* **18**, E221–E231 (2012). 21958195 10.1111/j.1469-0691.2011.03648.x

[31] Blaizot, R. et al. Outbreak of Amazonian toxoplasmosis: A one health investigation in a remote amerindian community. *Front. Cell. Infect. Microbiol.* **10**, 401 (2020). 33042853 10.3389/fcimb.2020.00401 PMC7516351

[32] Cortés, D. A. et al. Severe acute multi-systemic failure with bilateral ocular toxoplasmosis in immunocompetent patients from urban settings in Colombia: Case reports. *Am. J. Ophthalmol. Case Rep.* **18**, 100661 (2020). 32195446 10.1016/j.ajoc.2020.100661 PMC7078491

[33] Pena, H. F. J. et al. Toxoplasma gondii isolated from a Brazilian patient with rare pulmonary toxoplasmosis has a novel genotype and is closely related to Amazonian isolates. *Parasitol. Res.* **120**, 1109–1113 (2021). 33420622 10.1007/s00436-020-07008-4

[34] Seguela, J. P., Larrouy, G., Serie, C. & Plenet, J. Toxoplasmose en Guyane Française. *Méd. Mal. Infect.* **5**, 546–548 (1975).

[35] Demar, M. et al. Fatal outbreak of human toxoplasmosis along the maroni river: Epidemiological, clinical, and parasitological aspects. *Clin. Infect. Dis.* **45**, e88–e95 (2007). 17806043 10.1086/521246

[36] Bahia-Oliveira, L. M., Boechat, M. S. B. & Peixe, R. G. Host immune response to Toxoplasma gondii and Ascaris lumbricoides in a highly endemic area: evidence of parasite co-immunomodulation properties influencing the outcome of both infections. 10.1590/s0074-02762009000200021 19430653

[37] Gouda, M. A. et al. Association between breakthrough infection with COVID-19 and Toxoplasma gondii: A cross-sectional study. *Sci. Rep.* **13**, 17636 (2023). 37848511 10.1038/s41598-023-44616-3 PMC10582182

[38] Mart, D. Y. Tegumentary leishmaniasis and coinfections other than HIV. 10.1371/journal.pntd.0006125 PMC5832191 29494584

[39] Borbón, T. Y. et al. Coinfection with Leishmania major and Staphylococcus aureus enhances the pathologic responses to both microbes through a pathway involving IL-17A. *PLoS Negl. Trop. Dis.* **13**, e0007247 (2019). 31107882 10.1371/journal.pntd.0007247 PMC6527190

[40] Epelboin, L. et al. Is dengue and malaria co-infection more severe than single infections? A retrospective matched-pair study in French Guiana. *Malar. J.* **11**, 142 (2012). 22549018 10.1186/1475-2875-11-142 PMC3403992

[41] Degarege, A. Plasmodium falciparum and soil-transmitted helminth co-infections among children in sub-Saharan Africa: A systematic review and meta-analysis. (2016). 10.1186/s13071-016-1594-2 PMC4908807 27306987

[42] Couppie, P. et al. Comparative study of cutaneous leishmaniasis in human immunodeficiency virus (HIV)-infected patients and non-HIV-infected patients in French Guiana. *Br. J. Dermatol.* **151**, 1165–1171 (2004). 15606511 10.1111/j.1365-2133.2004.06226.x

[43] Abbate, J. L. et al. Disentangling complex parasite interactions: Protection against cerebral malaria by one helminth species is jeopardized by co-infection with another. *PLoS Negl. Trop. Dis.* **12**, 1–13 (2018). 10.1371/journal.pntd.0006483 PMC5963812 29746467

[44] Douine, M. et al. Zoonoses and gold mining: A cross-sectional study to assess yellow fever immunization, Q fever, leptospirosis and leishmaniasis among the population working on illegal mining camps in French Guiana. *PLoS Negl. Trop. Dis.* **16**, e0010326 (2022). 35969647 10.1371/journal.pntd.0010326 PMC9410546

[45] Fogang, B. et al. High prevalence of asymptomatic malarial anemia and association with early conversion from asymptomatic to symptomatic infection in a plasmodium falciparum hyperendemic setting in Cameroon. *Am. J. Trop. Med. Hyg.* **106**, 293–302 (2022). 10.4269/ajtmh.21-0316 PMC8733519 34724628

[46] Sáenz, F. E. et al. Malaria epidemiology in low-endemicity areas of the northern coast of Ecuador: High prevalence of asymptomatic infections. *Malar. J.* **16**, 300 (2017). 28747199 10.1186/s12936-017-1947-0 PMC5530496

[47] Vallejo, A. F. et al. High prevalence of sub-microscopic infections in Colombia. *Malar. J.* **14**, 201 (2015). 25971594 10.1186/s12936-015-0711-6 PMC4438632

[48] Mosnier, E. et al. Prevalence of Plasmodium spp. in the Amazonian Border Context (French Guiana–Brazil): Associated Factors and Spatial Distribution. *Am. J. Trop. Med. Hyg.* **102**, 130–141 (2020). 31769403 10.4269/ajtmh.19-0378 PMC6947805

[49] Sagna, A. B. et al. Plasmodium falciparum infection during dry season: IgG responses to Anopheles gambiae salivary gSG6-P1 peptide as sensitive biomarker for malaria risk in Northern Senegal. *Malar. J.* **12**, 301 (2013). 23988032 10.1186/1475-2875-12-301 PMC3766161

[50] Almeida, G. G. et al. Asymptomatic Plasmodium vivax malaria in the Brazilian Amazon: Submicroscopic parasitemic blood infects Nyssorhynchus darlingi. *PLoS Negl. Trop. Dis.* **15**, e0009077 (2021). 34714821 10.1371/journal.pntd.0009077 PMC8555776

[51] Babekir, A. et al. The Association of Toxoplasma gondii IgG and Liver Injury in US Adults. *IJERPH* **19**, 7515 (2022). 35742764 10.3390/ijerph19127515 PMC9223808

[52] Bi, D. et al. Clinical characteristics of platelet-mediated killing circulating parasite of major human malaria. *Ann. Med.* **55**, 2221453 (2023). 37310126 10.1080/07853890.2023.2221453 PMC10266116

[53] Prakash, D. et al. Clusters of cytokines determine malaria severity in *Plasmodium falciparum–* infected patients from endemic areas of Central India. *J. Infect. Dis.* **194**, 198–207 (2006). 16779726 10.1086/504720

[54] Herbert, F. et al. Evidence of IL-17, IP-10, and IL-10 involvement in multiple-organ dysfunction and IL-17 pathway in acute renal failure associated to Plasmodium falciparum malaria. *J. Transl Med.* **13**, 369 (2015). 26602091 10.1186/s12967-015-0731-6 PMC4658812

[55] Simon, S. *Toxoplasmose amazonienne: Biodiversité de Toxoplasmagondii chez l’homme et l’animal, conséquences pathologiques etmécanismes de virulence* (Université de Guyane, 2018).

[56] Sasai, M., Pradipta, A. & Yamamoto, M. Host immune responses to *Toxoplasma gondii*. *Int. Immunol.* **30**, 113–119 (2018). 29408976 10.1093/intimm/dxy004

[57] Ivanova, D. L. et al. Innate lymphoid cells in protection, pathology, and adaptive immunity during apicomplexan infection. *Front. Immunol.* **10**, 196 (2019). 30873151 10.3389/fimmu.2019.00196 PMC6403415

[58] Schmitz, J. et al. IL-33, an Interleukin-1-like Cytokine that Signals via the IL-1 Receptor-Related Protein ST2 and Induces T Helper Type 2-Associated Cytokines. *Immunity* **23**, 479–490 (2005). 16286016 10.1016/j.immuni.2005.09.015

[59] Saidi, N. et al. Clinical and immunological spectra of human cutaneous leishmaniasis in North Africa and French Guiana. *Front. Immunol.* **14**, 1134020 (2023). 37575260 10.3389/fimmu.2023.1134020 PMC10421664

[60] Mesquita, T. G. R. D. et al. Distinct plasma chemokines and cytokines signatures in Leishmania guyanensis-infected patients with cutaneous leishmaniasis. *Front. Immunol.* **13**, 974051 (2022). 36091007 10.3389/fimmu.2022.974051 PMC9453042

[61] Baratta-Masini, A. Mixed cytokine profile during active cutaneous leishmaniasis and in natural resistance. *Front. Biosci.* **12**, 839 (2007). 17127341 10.2741/2106

[62] Mendonça, L. S. O. et al. Characterization of serum cytokines and circulating microRNAs that are predicted to regulate inflammasome genes in cutaneous leishmaniasis patients. *Exp. Parasitol.* **210**, 107846 (2020). 32001303 10.1016/j.exppara.2020.107846

[63] Blakytny, R. et al. Latent TGF-beta1 activation by platelets. *J. Cell. Physiol.* **199**, 67–76 (2003). 10.1002/jcp.10454 14978736

[64] Korniluk, A., Koper-Lenkiewicz, O. M., Kamińska, J. & Kemona, H. Dymicka-Piekarska, V. Mean platelet volume (MPV): New perspectives for an old marker in the course and prognosis of inflammatory conditions. *Mediat. Inflamm.* **2019**, 1–14 (2019). 10.1155/2019/9213074 PMC6501263 31148950

[65] An, I. et al. Evaluation of inflammatory parameters in patients with cutaneous leishmaniasis. *Dermatol. Therapy* **34**, (2021). 10.1111/dth.14603 33249697

[66] Guedes, K. S., Sanchez, B. A. M., Gomes, L. T. & Fontes, C. J. F. Aspartate aminotransferase-to-platelet ratio index (APRI): A potential marker for diagnosis in patients at risk of severe malaria caused by Plasmodium vivax. *PLoS ONE*. **14**, e0224877 (2019). 31765438 10.1371/journal.pone.0224877 PMC6876935

[67] Tovar Acero, C. et al. IL-4, IL-10, CCL2 and TGF-β as potential biomarkers for severity in Plasmodium vivax malaria. *PLoS Negl. Trop. Dis.* **16**, e0010798 (2022). 36178979 10.1371/journal.pntd.0010798 PMC9555658

[68] Bueno, L. L., Morais, C. G., Lacerda, M. V., Fujiwara, R. T. & Braga, É. M. Interleukin-17 producing T helper cells are increased during natural Plasmodium vivax infection. *Acta Trop.* **123**, 53–57 (2012). 22476130 10.1016/j.actatropica.2012.02.071

[69] Pascual-Reguant, A. et al. TH17 cells express ST2 and are controlled by the alarmin IL-33 in the small intestine. *Mucosal Immunol.* **10**, 1431–1442 (2017). 28198366 10.1038/mi.2017.5

[70] Komai-Koma, M. et al. Interleukin-33 promoting Th1 lymphocyte differentiation dependents on IL-12. *Immunobiology* **221**, 412–417 (2016). 26688508 10.1016/j.imbio.2015.11.013 PMC4731778

[71] Settles, E. W., Moser, L. A., Harris, T. H. & Knoll, L. J. Toxoplasma gondii Upregulates Interleukin-12 to prevent plasmodium berghei-induced experimental cerebral malaria. *Infect. Immun.* **82**, 1343–1353 (2014). 24396042 10.1128/IAI.01259-13 PMC3957979

[72] Lee, D. H., Chu, K. B., Kang, H. J., Lee, S. H. & Quan, F. S. Previous infection with plasmodium berghei confers resistance to toxoplasma gondii infection in mice. *Kor. J. Parasitol.* **57**, 93–99 (2019). 31104401 10.3347/kjp.2019.57.2.93 PMC6526213

[73] Douine, M. et al. Self-diagnosis and self-treatment of malaria in hard-to-reach and mobile populations of the Amazon: Results of Malakit, an international multicentric intervention research project. *Lancet Reg. Health - Americas*. **4**, 100047 (2021). 36776708 10.1016/j.lana.2021.100047 PMC9903903

[74] Reller, M. E., Chen, W. H., Dalton, J., Lichay, M. A. & Dumler, J. S. Multiplex 5′ nuclease quantitative real-time PCR for clinical diagnosis of malaria and species-level identification and epidemiologic evaluation of malaria-causing parasites, including plasmodium knowlesi. *J. Clin. Microbiol.* **51**, 2931–2938 (2013). 23804387 10.1128/JCM.00958-13 PMC3754650

[75] World Health Organization. *World Malaria Report 2012* (World Health Organization, 2012).

[76] World Health Organization. Severe Malaria. *Trop. Med. Int. Health*. **19**, 7–131 (2014). 25214480 10.1111/tmi.12313_2

[77] Ashley, E. A., Pyae Phyo, A., Woodrow, C. J. & Malaria *Lancet* **391**, 1608–1621 (2018). 29631781 10.1016/S0140-6736(18)30324-6

[78] Simon, S. et al. Cutaneous leishmaniasis in French Guiana: Revising epidemiology with PCR-RFLP. *Trop. Med. Health*. **45**, 5 (2017). 28265182 10.1186/s41182-017-0045-x PMC5331739

[79] Breiman, L., Friedman, J., Olshen, R. A. & Stone, C. J. *Classification and Regression Trees*. (1984).

