# Supplementary material for: Unravelling complex interactions during Toxoplasma, Plasmodium, and Leishmania co-infections in French Guiana
Source: Sci Rep. 2026 Mar 16;16:13717. doi: 10.1038/s41598-026-40930-8 (PMC13125212; doi:10.1038/s41598-026-40930-8)
Supplement: Supplementary file 1 — Supplementary Material 1 [file 41598_2026_40930_MOESM1_ESM.pdf]

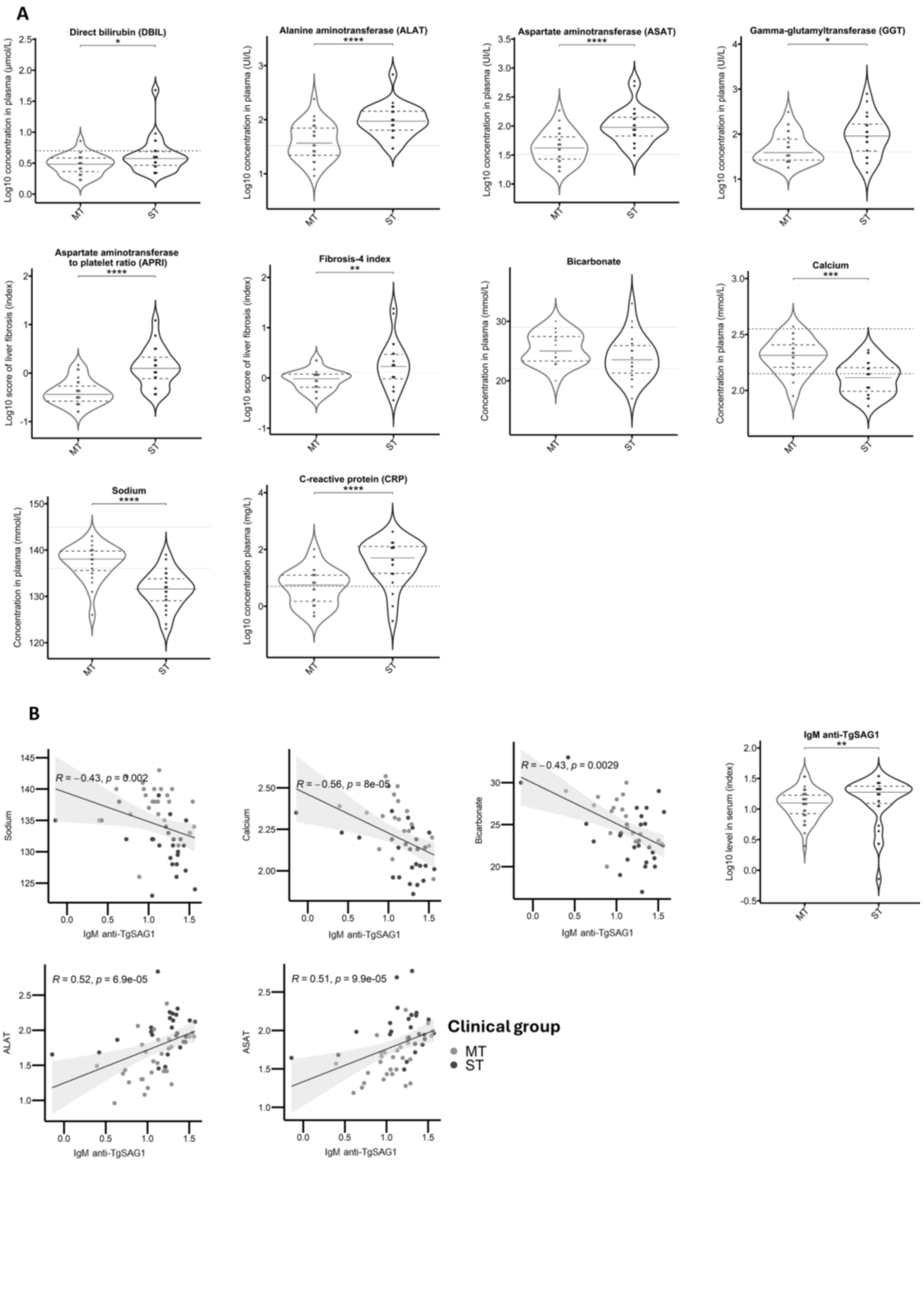

A
Direct bilirubin (DBIL)
*
Log10 concentration in plasma (µmol/L)
Alanine aminotransferase (ALAT)
****
Log10 concentration in plasma (UI/L)
Aspartate aminotransferase (ASAT)
****
Log10 concentration in plasma (UI/L)
Gamma-glutamyltransferase (GGT)
*
Log10 concentration in plasma (UI/L)
Aspartate aminotransferase to platelet ratio (APRI)
****
Log10 score of liver fibrosis (index)
Fibrosis-4 index
**
Log10 score of liver fibrosis (index)
Bicarbonate
Concentration in plasma (mmol/L)
Calcium
***
Concentration in plasma (mmol/L)
Sodium
****
Concentration in plasma (mmol/L)
C-reactive protein (CRP)
****
Log10 concentration plasma (mg/L)
MT
ST
B
R = −0.43, p = 0.002
Sodium
IgM anti-TgSAG1
R = −0.56, p = 8e-05
Calcium
IgM anti-TgSAG1
R = −0.43, p = 0.0029
Bicarbonate
IgM anti-TgSAG1
IgM anti-TgSAG1
**
Log10 level in serum (index)
R = 0.52, p = 6.9e-05
ALAT
IgM anti-TgSAG1
R = 0.51, p = 9.9e-05
ASAT
IgM anti-TgSAG1
Clinical group
MT
ST

Supplementary Fig. S1. Discriminant biochemical factors in clinical groups of toxoplasmosis patients. A Violin plots show the median and the quartiles. The dashed lines indicate the standard clinical reference values for each variable. Each point corresponds to an individual. B. Linear regression between IgM anti-*Tg*SAG1, a diagnosis marker of AT and biochemical factors with correlation of Spearman. Individual point was coloured in accordance with the sub-phenotype group and confidence interval was showed in grey. Only the correlations with $p < 0.05$ are shown and the coefficient of correlation is noted.

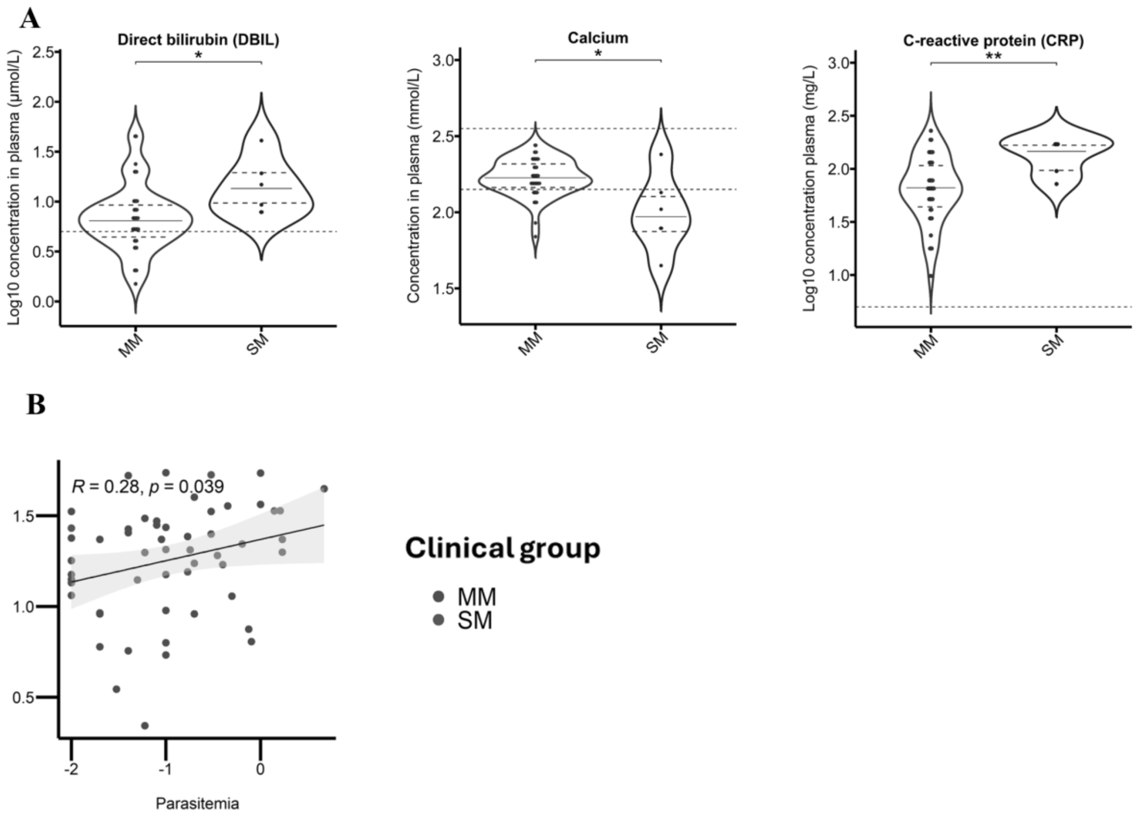

Supplementary Fig. S2. Discriminant biochemical factors in clinical outcome of malaria groups. **A** Violin plots show the median and the quartiles. The dashed lines indicate the standard clinical reference values for each variable. Each point corresponds to an individual. **B**. Linear regression between Parasite load, the diagnosis marker of MAL and biochemical factors with correlation of Spearman. Individual point was coloured in accordance with the sub-phenotype group and confidence interval was showed in grey. Only the correlations with $p < 0.05$ are shown and the coefficient of correlation is noted.

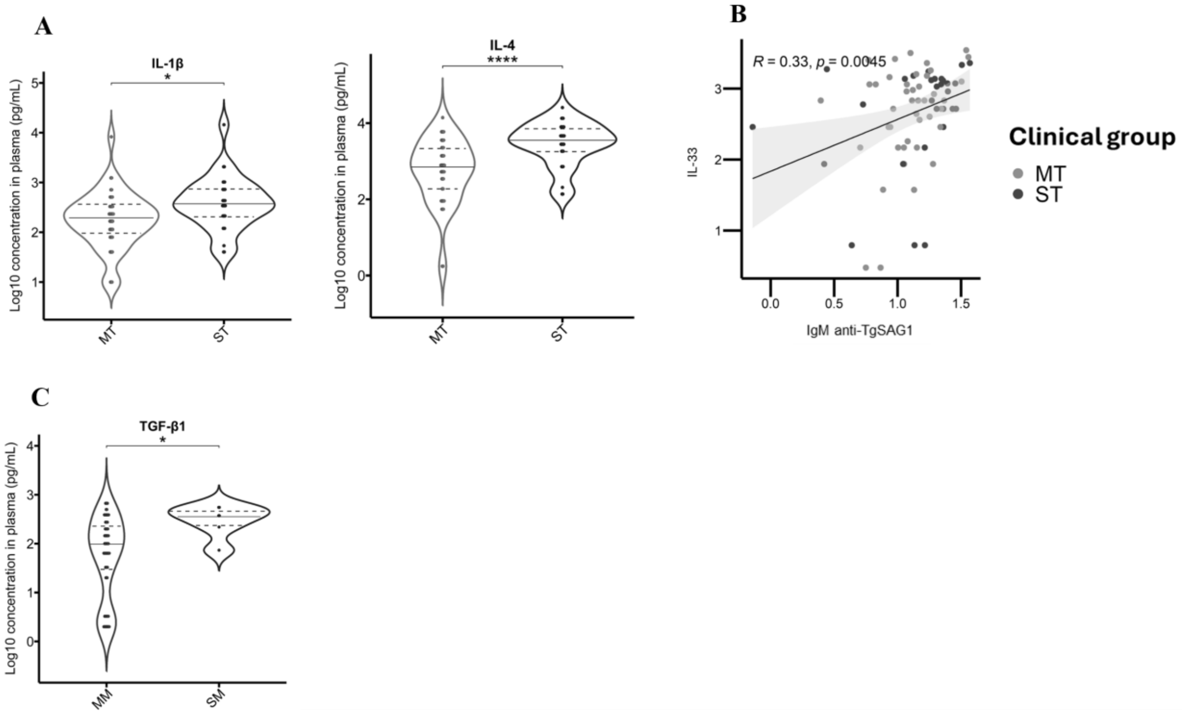

Supplementary Fig. S3. Discriminant cytokines in clinical outcome of disease groups**. A & C** Violin plots show the median and the quartiles. The dashed lines indicate the standard clinical reference values for each variable. Each point corresponds to an individual. **B**. Linear regression between IgM anti-*Tg*SAG1 and cytokines with correlation of Spearman analysis. Individual point was coloured in accordance with the sub-phenotype group and confidence interval was showed in grey. Only the correlations with $p < 0.05$ are shown and the coefficient of correlation is noted. **A-B** analyses in AT group and **C** in MAL group.

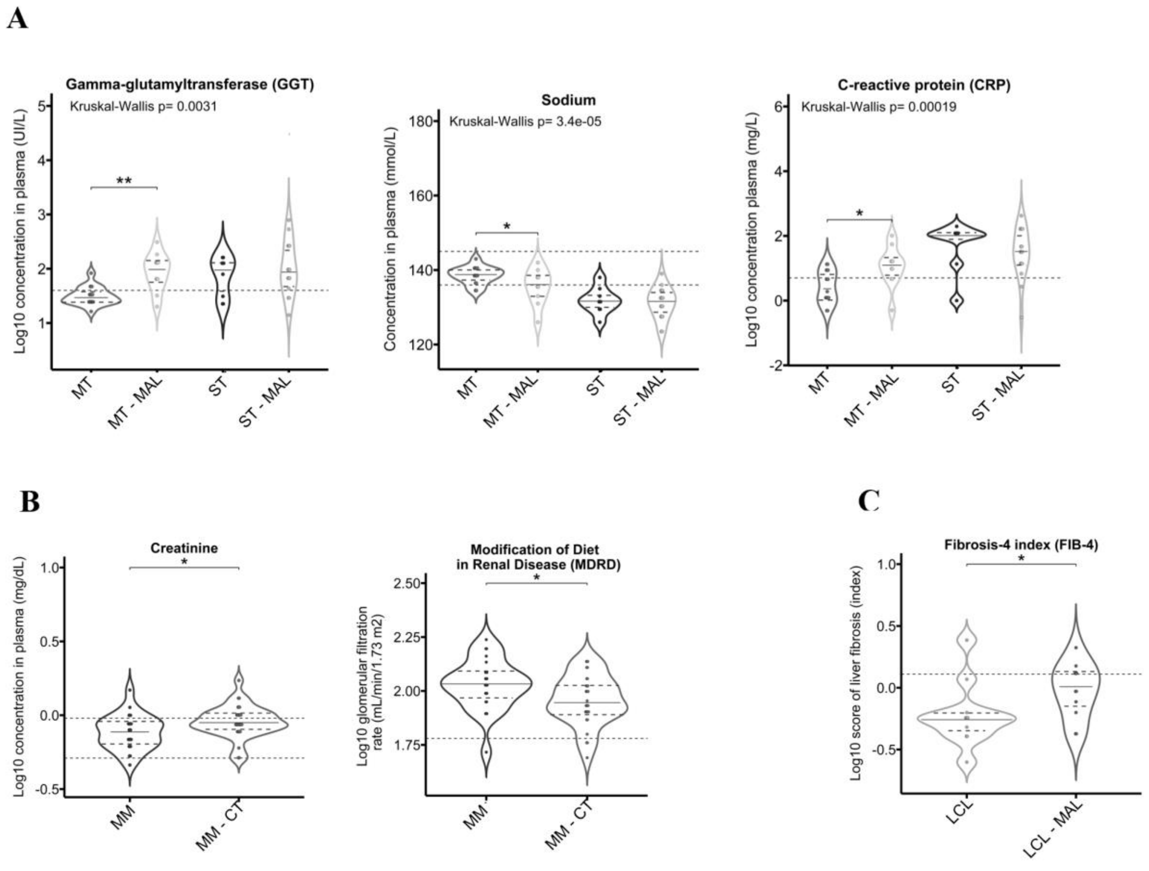

Supplementary Fig. S4. Discriminant biochemical variables in the groups positive to protozoan multi-infections in accordance with severity. Violin plots show the median and the quartiles. The dashed lines indicate the standard clinical reference values for each variable. Each point corresponds to an individual. **A** Comparison in toxoplasmosis subgroups with a history of malaria. **B** Comparison into MM subgroup coinfected or not with *T. gondii*. **C** Comparison in LCL subgroup with history malaria or not independently of past infection with *T. gondii*. Significance was determined using Conover post-hoc test (groups >2) or Wilcoxon test (groups =2). The significance level is denoted as: '**': $p < 0.01$; '*': $p < 0.05$

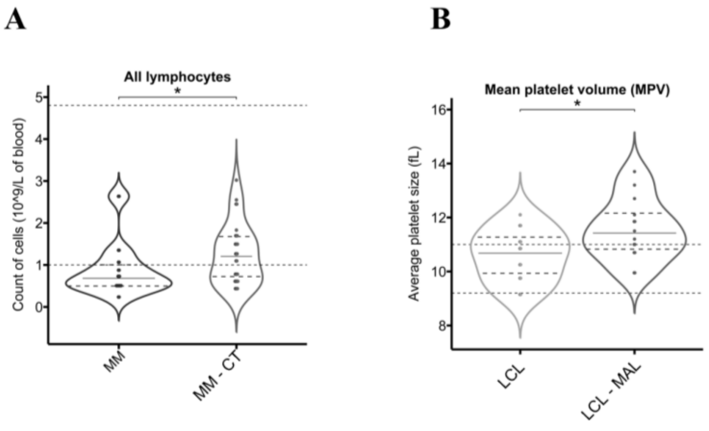

Supplementary Fig. S5. Discriminant cellular and constant blood variables in the groups positive to protozoan multi-infections in accordance with severity. Violin plots show the median and the quartiles. The dashed lines indicate the standard clinical reference values for each variable. Each point corresponds to an individual. **A** Comparison into MM subgroup coinfected or not with *T. gondii*. **B** Comparison in LCL subgroup with a history malaria or not independently of past infection with *T. gondii*. The significance level using Wilcoxon test is denoted as: ‘*’p < 0.05.

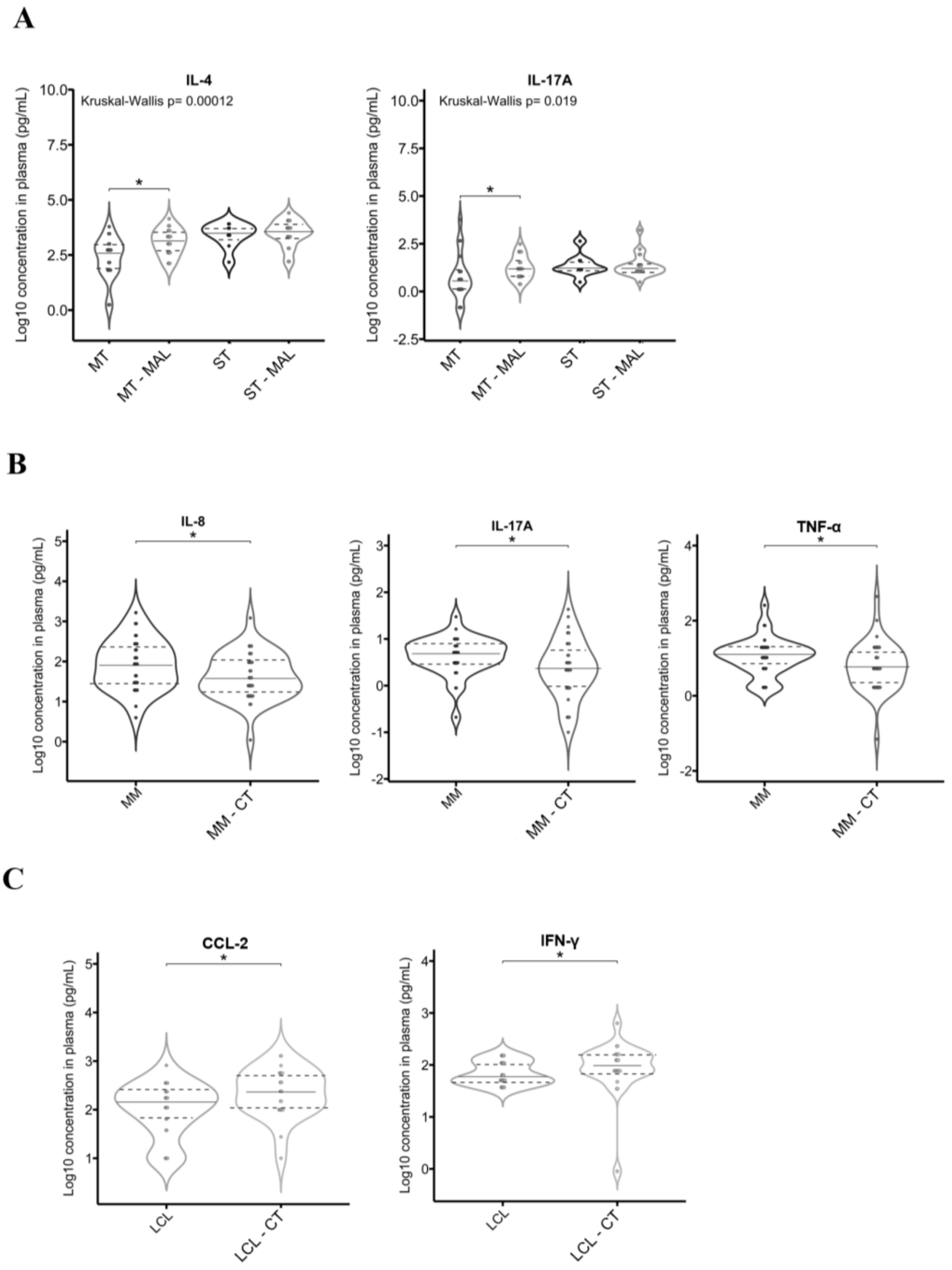

Supplementary Fig. S6. Discriminant cytokine and chemokine in the groups positive to protozoan multi-infections in accordance with severity. Violin plots show the median and the quartiles. The dashed lines indicate the standard clinical reference values for each variable. Each point corresponds to an individual. **A** Comparison in toxoplasmosis subgroups with a history of malaria. **B** Comparison into MM subgroup coinfected or not with *T. gondii*. **C** Comparison in LCL subgroup with a history malaria or not independently of past infection with *T. gondii*. Significance was determined using Conover post-hoc test (groups >2) or Wilcoxon

test (groups =2). The significance level using test is denoted as: ‘*’$p < 0.05$.
