# Supplementary material for: Unravelling complex interactions during Toxoplasma, Plasmodium, and Leishmania co-infections in French Guiana
Source: Sci Rep. 2026 Mar 16;16:13717. doi: 10.1038/s41598-026-40930-8 (PMC13125212; doi:10.1038/s41598-026-40930-8)
Supplement: Supplementary file 2 — Supplementary Material 2 [file 41598_2026_40930_MOESM2_ESM.pdf]

Supplementary Table S1. biochemical and cellular variables studied

Haematology	Blood biochemistry		
**Blood measurements**	**Electrolytes**	**Enzymology/liver function**	**Coagulation**
Haematocrit	Sodium	Aspartate aminotransferase (ASAT)	Prothrombin time
Haemoglobin	Potassium	Alanine aminotransferase (ALAT)	International normalized ration
Mean corpuscular haemoglobin concentration	Modification of diet in renal disease	Gamma glutamyl transferase (GGT)	Activated partial thromboplastin time
Mean cell haemoglobin	Chloride	Alkaline phosphatase	Fibrinogen
Mean platelet volume (MPV)	Total calcium	Direct bilirubin	
Mean corpuscular volume	Total proteins	Indirect bilirubin	
Red blood cell distribution width	Bicarbonate	Total bilirubin (TBIL)	
	Creatinine	Lactate dehydrogenase	
	Osmolarity	Fibrosis-4-index (FIB-4)	
		Aspartate aminotransferase-to-platelet ratio index (APRI)	
**Cells**	**Lipids and carbohydrates**	**Pathological markers**	
Platelets	Lipase	Creatinine phosphokinase	
Red blood cells	Plasma glucose	Cardiac troponin	
White blood cell count	Lactate	C-reactive protein (CRP)	
Lymphocytes			
Monocytes			
Basophils granulocytes			
Neutrophils granulocytes			
Eosinophils granulocytes			

Supplementary Table S2. Linear regression analysis

Variables	Covariables	Pr (>F)
Calcium	Gender	**0.0041**
	Age	0.0764
	T. gondii seropositivity	0.5188
	Psp seropositivity	0.4260
	Number of lesions	0.1972
Bicarbonate	Gender	**0.0003**
	Age	0.4331
	T. gondii seropositivity	0.1801
	P.sp seropositivity	0.8113
	Number of lesions	0.2288
Sodium	Gender	**0.0328**
	Age	0.7793
	T. gondii seropositivity	0.7485
	Psp seropositivity	0.6645
	Number of lesions	0.1805
Fibrosis-4-index	Gender	0.8722
	Age	**0.0001**
	T. gondii seropositivity	0.7139
	Psp seropositivity	**0.0354**
	Number of lesions	0.5049
CRP	Gender	**0.0028**
	Age	0.8082
	T. gondii seropositivity	0.0835
	Psp seropositivity	0.1926
	Number of lesions	0.7972
MPV	Gender	0.9879
	Age	0.5366
	T. gondii seropositivity	0.1197
	Psp seropositivity	**0.0185**
	Number of lesions	0.0508
